# Spatio-temporal optical coherence tomography provides full thickness imaging of the chorioretinal complex

**DOI:** 10.1016/j.isci.2022.105513

**Published:** 2022-11-05

**Authors:** Egidijus Auksorius, Dawid Borycki, Piotr Wegrzyn, Bartosz L. Sikorski, Kamil Lizewski, Ieva Zickiene, Mounika Rapolu, Karolis Adomavicius, Slawomir Tomczewski, Maciej Wojtkowski

**Affiliations:** 1 International Center for Translational Eye Research (ICTER), ul. Skierniewicka 10a, 01-230 Warsaw, Poland; 2 Center for Physical Sciences and Technology (FTMC), Saulėtekio al. 3, LT-10257 Vilnius, Lithuania; 3 Institute of Physical Chemistry, Polish Academy of Sciences, Kasprzaka 44/52, 01-224 Warsaw, Poland; 4 Faculty of Physics, University of Warsaw, Pasteura 5, 02-093 Warsaw, Poland; 5 Department of Ophthalmology, Nicolaus Copernicus University, Skłodowskiej-Curie 9, 85-090 Bydgoszcz, Poland; 6 Oculomedica, Eye Research & Development Center, Ogrody 14, 85-870 Bydgoszcz, Poland

**Keywords:** Medical imaging, Optical imaging, Methodology in biological sciences

## Abstract

Despite the rapid development of optical imaging methods, high-resolution *in vivo* imaging with penetration into deeper tissue layers is still a major challenge. Optical coherence tomography (OCT) has been used successfully for non-invasive human retinal volumetric imaging *in vivo*, advancing the detection, diagnosis, and monitoring of various retinal diseases. However, there are important limitations of volumetric OCT imaging, especially coherent noise and the limited axial range over which high resolution images can be acquired. The limited range prevents simultaneous measurement of the retina and choroid with adequate lateral resolution. In this article, we address these limitations with a technique that we term spatio-temporal optical coherence tomography (STOC-T), which uses light with controlled spatial and temporal coherence and advanced signal processing methods. STOC-T enabled the acquisition of high-contrast and high-resolution coronal projection images of the retina and choroid at arbitrary depths.

## Highlights

• STOC-T enables uniform 5 μm resolution imaging of the chorioretinal complex
• Full-field detection and digital defocus correction ensure >1 mm axial imaging range
• High contrast coronal projections of choroid are obtained for selected depths
• Quantitative morphometry metrics are derived from choriocapillaris image

## Graphical abstract

**Figure:**
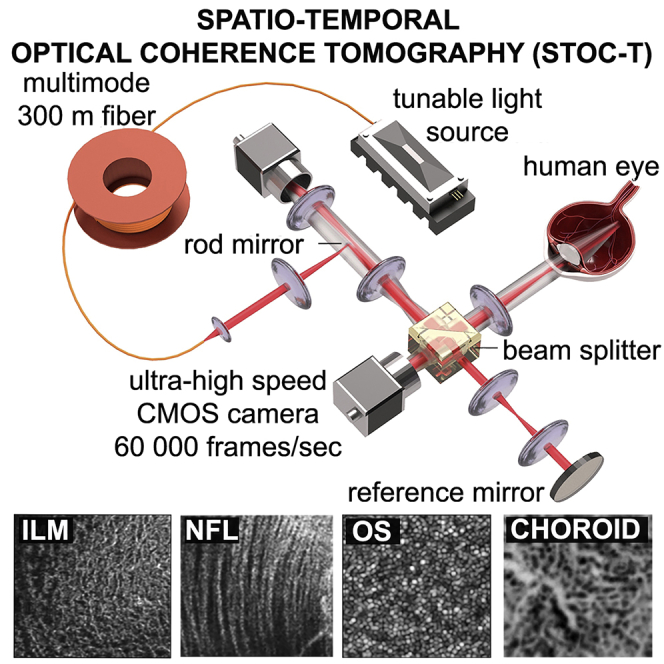

## Introduction

Optical imaging technologies have significantly improved visualization of retinal pathophysiology and have had a substantial impact on basic and translational medical research. These technologies, such as scanning laser ophthalmoscopy, and fluorescein and indocyanine green (ICG) angiographies have translated into more accurate diagnosis, and improved management of numerous diseases of the eye. Optical coherence tomography (OCT)^1^^,^^2^ has become a standard of care for diagnosing and monitoring the treatment of such retinal diseases as age-related macular degeneration (AMD), diabetic retinopathy (DR), and glaucoma,^3^^,^^4^^,^^5^^,^^6^^,^^7^ which are among the leading causes of vision loss in the world. Despite rapid development of OCT technology, it is still challenging for classical OCT techniques to distinguish important morphological elements. Although OCT angiography (angio-OCT) significantly advanced the field of OCT by allowing visualization of the retinal and choroidal microvasculature without the injection of exogenous dyes,^8^^,^^9^^,^^10^ the quality of the angiographic images is still limited by the optics and hardware, to the same degree that morphological OCT images are. The hard-to-image choroid of the human eye plays a key role in the proper function of the retina by nourishing its outer layers.^11^ The choriocapillaris (CC), retinal pigmented epithelium (RPE) and photoreceptor cells form a unitary metabolic complex making CC a very important element in the pathophysiology of retinal diseases, including AMD, DR, and glaucoma.^12^^,^^13^^,^^14^^,^^15^ The best currently known method for visualization of choroid vessels is ICG angiography. This method, because of its time selectivity, allows efficient filtering of signals coming from tissues other than blood vessels and allows observation of choroid projections in short times after administration of the contrast dye. However, ICG does not allow axial sectioning so that individual layers of the choroid can be observed. It is also not possible to image CC with ICG.

Imaging of the CC has remained a quintessential challenge of OCT because of its dense structure with short intercapillary distances (ICDs), and interference from the pigmentation of the RPE layer and choroidal melanocytes that strongly absorb and scatter light. Numerous efforts have been made to use commercially available OCT devices for choroidal imaging.^16^^,^^17^ However, with an ICD size of 5–20 μm, devices with 15–20 μm resolution provide only rather poor imaging quality. Nevertheless, angio-OCT has been used extensively to image the CC^17^^,^^18^^,^^19^ but, unlike the retinal microvasculature, the CC has a much denser capillary network and provides fewer signals. For instance, it was recently found that angio-OCT images acquired at different choroidal depths are similar in appearance, suggesting that images of the CC might be projected onto other layers, such as Sattler’s layer, which would explain the reported observation of a dense network at depths greater than 20 μm from the RPE.^20^ Such lack of specificity between different choroidal layers makes quantitative analysis meaningless. There have also been many attempts to image the choroid and choriocapillaris with experimental OCT systems.^21^^,^^22^^,^^23^ The most interesting results were obtained using ultrafast imaging with swept-source OCT at 1060 nm where a decent compromise between imaging depth and lateral resolution could be achieved.^24^^,^^25^^,^^26^ Impressive reconstructions of choriocapillaries were obtained in this work, but lateral resolution remains a major limitation in reconstructing the morphology of the chorioretinal complex at different depths.

**Figure 1:**
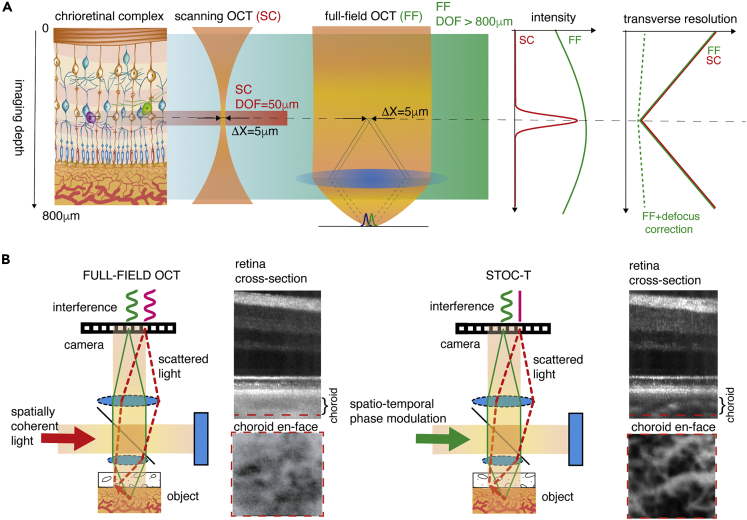
Concept of chorioretinal imaging with STOC-T (A) In STOC-T, the retina is probed with complex spatiotemporal light fields according to a wide-field imaging scheme (Full-Field OCT). As a result, there is no loss of light intensity with applied defocus, as is the case with confocal detection in scanning OCT methods. However, there is still image blurring associated with defocus. This blur, however, can be removed by the numerical defocus correction provided by the signals obtained with high-speed full-field interferometric registration. This preserves high (5um) lateral resolution with significant imaging depth, covering the entire extent of the chorioretinal complex (800um). (B) The complex spatiotemporal light fields guarantee the removal of the optical cross-talk effect created by scattered light in the sample. In STOC-T, scattered light that hits the camera pixels without recovering the original structure does not contribute to interference signals and thus to the volumetric reconstruction.

Fourier domain, a scanning OCT method that can be found in most commercially available OCT instruments is present in two versions: SD-OCT and Swept-Source OCT.^27^ In both cases the scanning OCT systems achieve high axial resolution with two orders of magnitude larger axial imaging range, but at the expense of poorer lateral resolution. The reason for the compromised resolution is fundamental because the axial imaging range in scanning OCT determines both the sharpness and the intensity of images, because of the confocal parameter (Figure 1A). In such case the axial imaging range varies as a function of $\Delta x^{2}$, where Δx is lateral resolution; therefore, it quickly narrows down with better lateral resolution.^28^ Furthermore, motion artifacts, speckle noise, and ocular aberrations further compromise the image quality, which becomes insufficient for CC imaging.^29^ Adaptive optics (AO) OCT,^30^ which corrects for ocular aberrations, can address the resolution as well as the speckle size problem. Specifically, AO-OCT achieves the best possible lateral resolution and, consequently, the smallest speckle size by imaging through a dilated pupil (of ∼8 mm in diameter). For example, AO-OCT was able to visualize the choriocapillaris with 2.4 μm lateral resolution, allowing some preliminary morphometry to be performed on the acquired images.^31^^,^^32^ However, improved lateral resolution further restricts the already-restricted axial range. For instance, the field of view (FOV) in the above-mentioned example was limited to ∼0.3 mm × 0.15 mm and the axial range to only ∼30 μm,^31^ which is insufficient to collect backscattered light (with adequate lateral resolution) from the entire retinal thickness, including the choroid, which is approximately 450 μm thick.^33^ Finally, the current high complexity of the AO-OCT system, with its small measurement range and long measurement time, limits this technology from being widely adopted in clinical practice. In Fourier domain Full-field OCT (FD-FF-OCT)^34^^,^^35^^,^^36^ the axial imaging range, also called depth of focus (DOF), does not depend on the Rayleigh range (confocal parameter) of a focused beam but rather on the Rayleigh range of the (collimated) illumination beam going onto the sample and reference mirror (Figure 1A). Interference fringes in such an interferometer will be formed over the extended axial range of the OCT images; however, images will be blurred (out of focus) in all the planes except the very focus. Nevertheless, it has been shown previously that because of the high phase stability of the FD-FF-OCT method, numerical defocus compensation can deblur images over a large axial range.^37^

However, despite the available large DOF, a fundamental limitation of the FD-FF-OCT technique has been the presence of optical crosstalk,^38^ which becomes worse deeper into the tissue, effectively limiting the achievable imaging depth. A rotating diffuser has been used in the past to remove the crosstalk,^34^^,^^39^ which however featured limited axial imaging range. We have recently demonstrated efficient crosstalk suppression in FD-FF-OCT that also features a long axial imaging range by using effective spatial phase randomization tools, such as a fast deformable membrane, and a long multimode optical fiber.^40^^,^^41^^,^^42^^,^^43^^,^^44^^,^^45^^,^^46^ Each of these tools enables control of the spatial coherence of the light to effectively eliminate cross-talk; and, with the condition of underfilling illumination pupils in both interferometric arms, they can make imaging with high DOF possible while maintaining high lateral resolution (Figure 1B). We call the new method spatio-temporal optical coherence tomography (STOC-T) to emphasize that the complex spatiotemporal light field (a combination of spatial and temporal coherence of light) defines its performance.

In this report, we show that retinal images acquired with the STOC-T system maintain uniform resolution of ∼5 μm in all three dimensions, over the entire thickness of about 800 μm, without any mechanical scanning. In addition, we demonstrate that STOC-T can acquire high-contrast volumetric chorioretinal images with reduced scattering effects. We have employed known data processing algorithms and developed new ones to handle and process the acquired data sets in order to produce highly-corrected 3D data (volumes) for retinas over large fields of view. The technology and algorithms used here enabled high transverse resolution imaging of the retina and the choroid at various depths, making visible for the first time the differentiation of morphology within the Sattler’s, Haller’s and choriocapillaris layers.

## Results

### DOF enhancement by STOC-T

**Figure 2:**
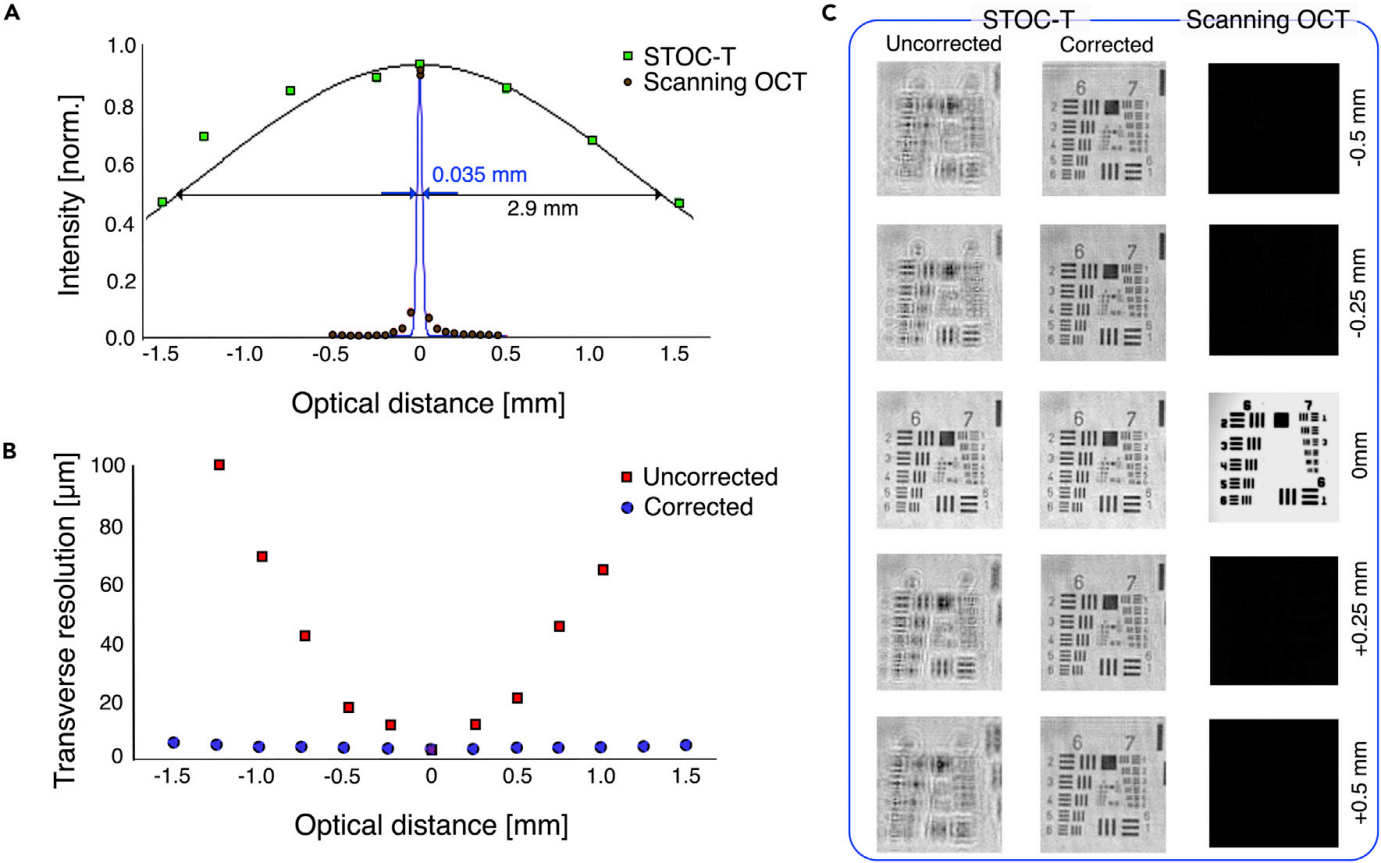
Measurement of DOF in STOC-T and scanning OCT, using a reflecting sample (USAF Test chart) (A–C) comparison of intensity attenuation for STOC-T and the scanning OCT, (B) effect of numerical defocus correction on transverse resolution loss in STOC-T, (C) Comparison of USAF test target images acquired with STOC-T and confocal scanning OCT at different defocus values.

To demonstrate the advantage of the STOC-T method over the classical scanning OCT techniques, first, a reflective sample (USAF resolution target) was imaged with STOC-T and scanning OCT systems. The sample was stepped along the optical axis and images were acquired at each (defocused) position, as shown in Figure 2. The data were then used to derive DOF curves for both techniques, as shown in the middle row of Figure 2, which demonstrates that the DOF for STOC-T is 2 orders of magnitude broader than that for the scanning OCT. Both systems had the same nominal lateral resolution value of 3.4 μm and used identical 10x objective lenses. The combination of the 10x objective and the USAF resolution target, which could be translated along the optical axis, could be considered as a model eye, where DOF could be measured more accurately than estimated in the human eye. For scanning OCT, the imaging DOF corresponds to the confocal parameter and has a fixed value of 35 μm for the given lateral resolution (3.4 μm). Therefore, signal that coming from 250 and 500 μm above or below the focal plane is only 1% and 0.5% of its maximum value, respectively, which corresponds to more than −40dB loss in signal visibility (Figures 2A and S11). In contrast, for STOC-T, the signal intensity value is attenuated to only 95% and 90%, respectively (for 250 μm and 500 μm above or below the focal plane), which corresponds to no more than -1dB of signal attenuation. Imaging the physically defocused USAF target with STOC-T clearly results in a blurred (defocused) image (Figure 2C); however, it can be computationally refocused and thereby brought back to the nominal resolution values (Figure 2B). Such computational image refocusing works over the entire imaging range of STOC-T (+-1.5 mm).

**Figure 3:**
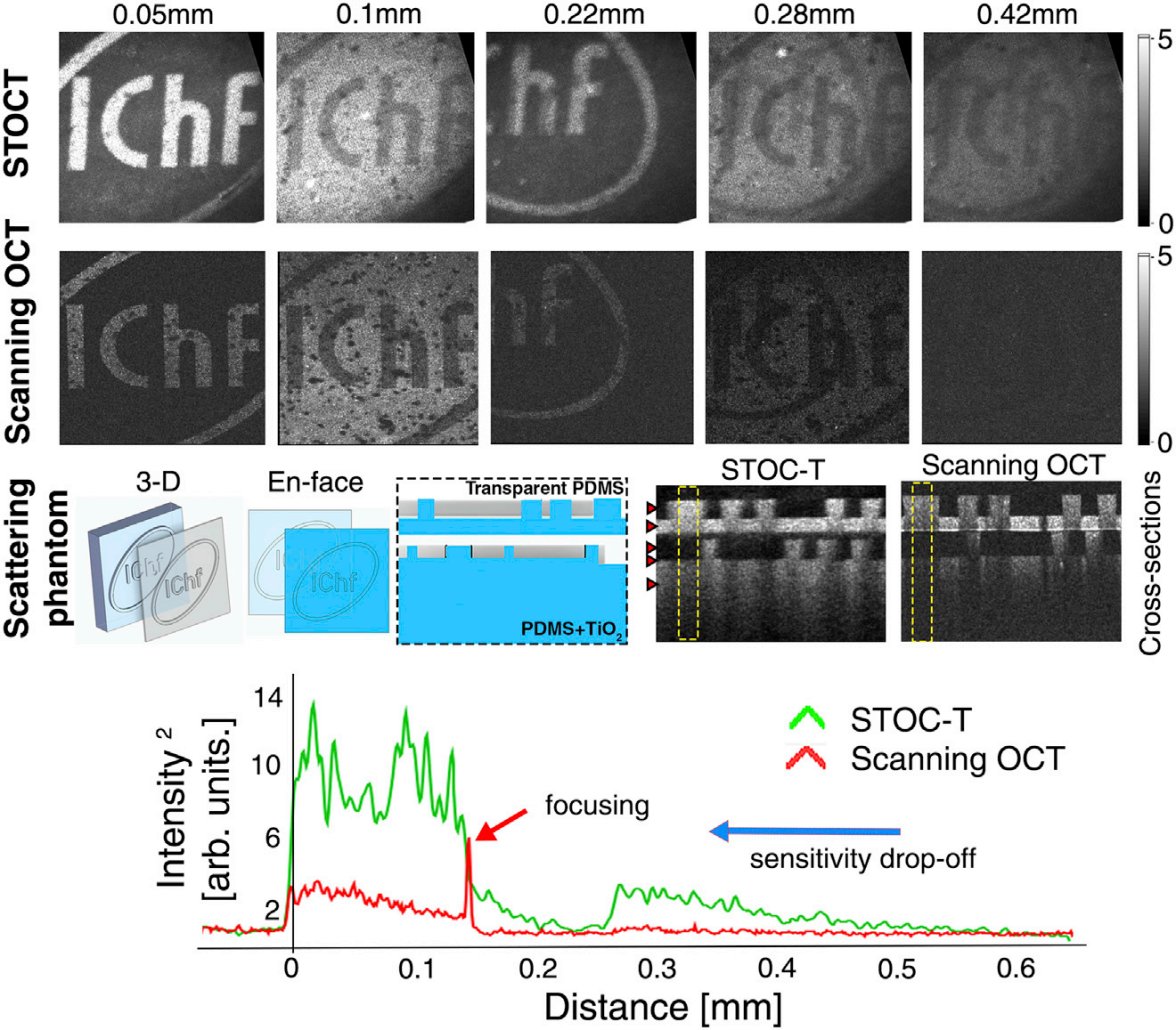
Signal decay as a function of depth in a volumetric scattering phantom Red arrows in the cross-sectional images denote the axial position of the *en face* images shown above. The curves were derived from the area indicated by the yellow dashed squares.

A strongly scattering phantom was also imaged with both systems to illustrate that the broader DOF in STOC-T would also lead to a broader axial imaging range in a real 3-D sample. For a controlled experiment on a scattering object, we used a volumetric bilayer phantom with a PDMS and TiO_2_ mixture as a scatterer.^41^ Although light is much more scattered in such a phantom than in the retina or choroid, we can use the phantom to validate the differences in imaging depth for scanning OCT and STOC-T at the same nominal value of lateral resolution (Figure 3). To quantitatively compare the differences in signal obtained by the two methods (featuring different sensitivities and illumination beam forming), we normalized the obtained volume reconstructions against the root-mean-square (RMS) of the noise. The signal decay curves as a function of depth (shown at the bottom of Figure 3) were derived from the cross-sectional images by integrating the signal (along the *x* axis) in the area marked by the yellow boxes. Consistent with the literature describing the behavior of the OCT signal when tightly focused in a scattering material,^47^^,^^48^ the curve for scanning OCT clearly shows the location of an elevated signal for the focal plane and a significantly attenuated signal for the rest of the scattering medium.

The signal from the deeper scattering layer is almost invisible. For the STOC-T technique, the signal from the object is visible throughout the 0.5 mm thickness and its extinction in depth is mainly related to the physical extinction processes in the sample. The *en face* reconstructions from different depths in the sample show a significant difference in signal strength between the two techniques. In order to minimize the effect of the sensitivity drop-off of the OCT scanning system, the measurement was done in the configuration when the zero optical path difference fell inside the phantom; i.e., the so-called enhanced depth imaging (EDI) configuration.^49^

### Improved choroidal penetration by STOC-T

**Figure 4:**
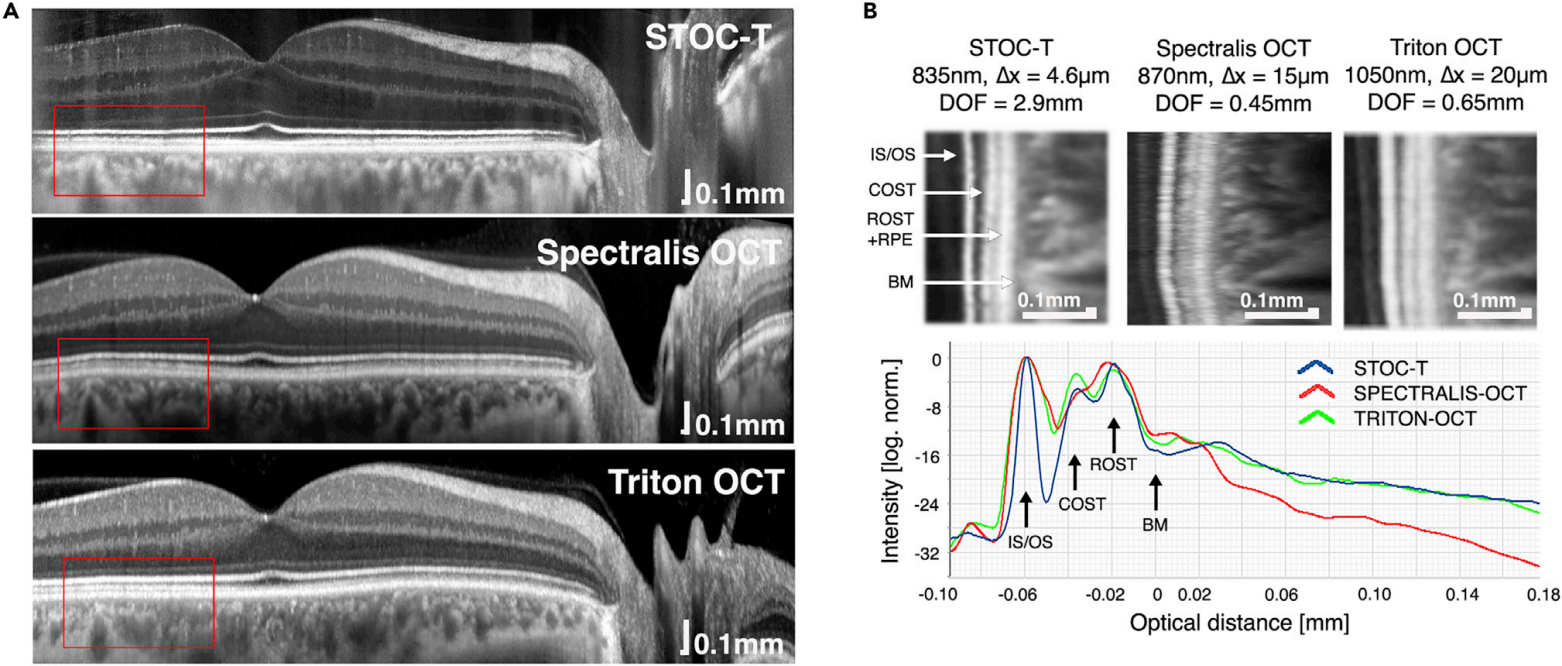
Comparison of the backscattered signal as a function of depth with STOC-T and two scanning OCT systems (Spectralis by Heidelberg Engineering and Triton by Topcon) (A and B) B-scans after averaging, (B) quantification of signal drop with imaging depth.

To show that the STOC-T system improves penetration depth into the choroid, we compared the relative changes in OCT signal against two commercially available scanning OCT devices with nominal values of lateral resolution of 15 μm and 20 μm, respectively, which correspond theoretically to values of the confocal parameter of 450 μm and 650 μm, respectively (Figure 4A).^50^ Because of differences in exposure times, number of averages, and scan densities, the signals were normalized with respect to the reflectance values from the IS/OS layer and the hypo-reflective signal corresponding to the outer parts of the photoreceptors. The graphs are shown on a logarithmic scale in Figure 4B. Despite having nearly 3–4 times higher lateral resolution, STOC-T has a DOF similar to that of the Triton OCT and significantly greater than that of the Spectralis OCT.

### Chorioretinal imaging and estimate of lateral resolution

**Figure 5:**
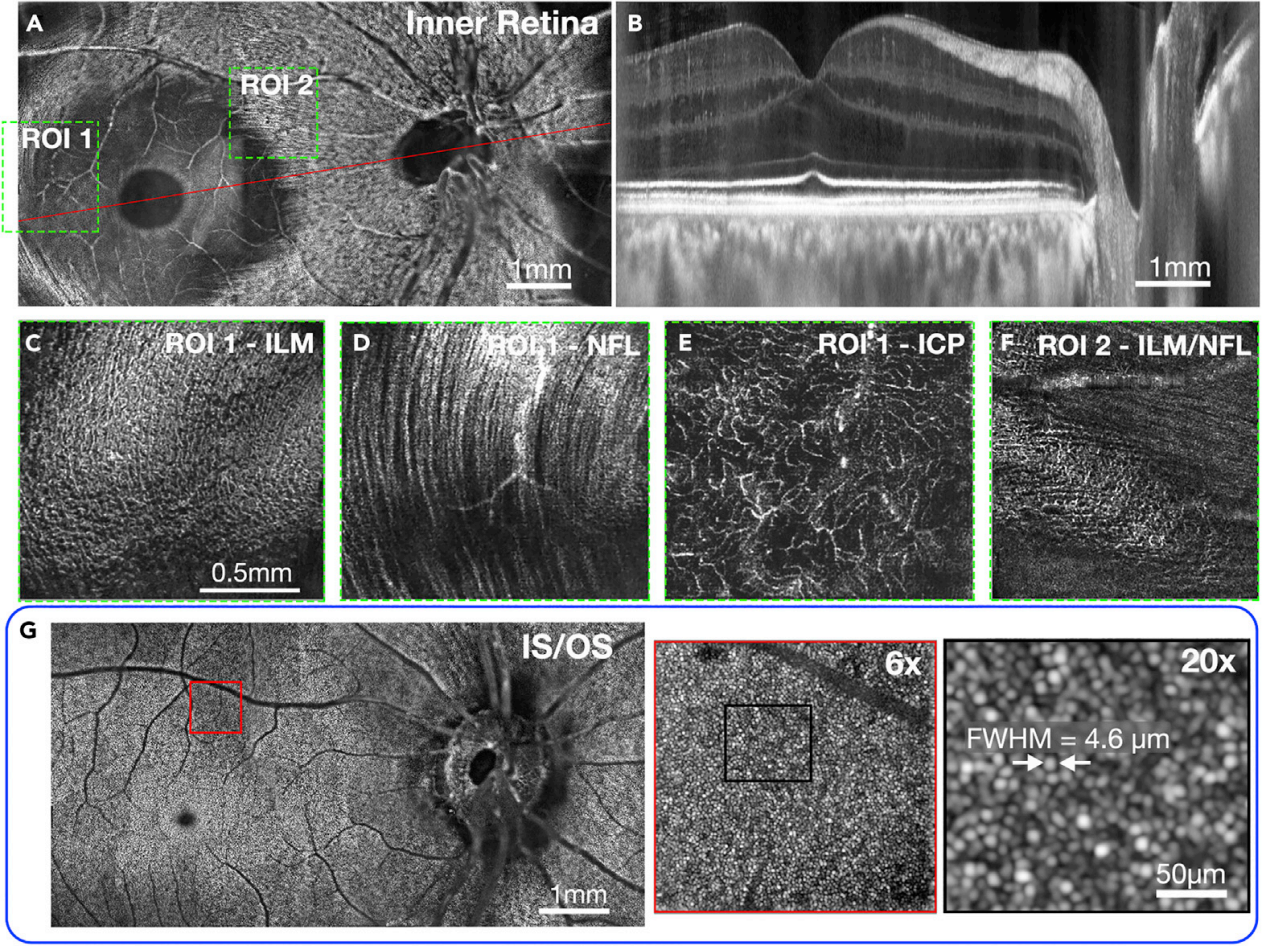
STOC-T imaging of retinal microstructure Montaged (stitched) retinal images (9 mm × 4.6 mm) of macula and optic nerve head; and panels with zoomed-in regions. (A) 15 μm *en face* projection of the inner retinal layers featuring the optic nerve discs, retinal nerve fiber layer (NFL), and retinal ganglion cell layer (GCL) in one image. (B) Cross-sectional image along the papillomacular axis of the human retina, indicated by red line in panel (A); (C–F) Details of the inner limiting membrane (ILM), NFL, and intermediate capillary plexus (ICP) are shown as thin layers (of 5 μm) and zoomed-in for the locations indicated by the dashed green boxes in (A); (G) STOC-T reconstruction of 15 μm thick coronal projection of the junction between inner and outer segments of photoreceptors (IS/OS); the red box in the IS/OS image is shown zoomed-in to various degrees on the right. The IS/OS reconstruction was numerically compensated for ocular aberrations revealing photoreceptor structure (22nd order Zernike polynomials).

Figure 5 demonstrates the capabilities of STOC-T when imaging the human retina *in vivo*. It shows *en face* (Figures 5A and 5G) and axial images (Figure 5B) over an extended field of view produced by stitching volumes/images in the lateral plane. To generate the images 30 vol were acquired per single imaging location of 1.7 mm × 1.7 mm in size, within 260 m sec. Stitching volumes captured from 32 adjacent locations produced a montage of images of 9 mm × 4.6 mm in size. STOC-T offers visualization of anatomical details of the retina in *en face* projection without the need for complex hardware solutions. Especially remarkable are the images of the structure of the thin Inner Limiting Membrane (ILM) that separates the nerve fiber layer (NFL) from the vitreous body. Figures 5C and 5F show the structure of this membrane, mapping the architecture of individual nerve fibers located just below it (shown in Figure 5D). In locations proximal to the optic disc, the ILM is more difficult to distinguish and the nerve fibers themselves begin to dominate.

Figure 5F shows transitioning between the ILM layer and nerve fibers. Structural reconstructions of anatomical retinal layers reveal superficial, deep, and intermediate capillary plexuses (ICP in Figure 5E) without the need for OCT angiography. With the additional use of a software-based technique of numerical aberration correction it is possible to optimize the lateral resolution to diffraction-limited and visualize the photoreceptor mosaic (Figure 5G).^46^ Imaging the photoreceptors allowed us to evaluate the effective value of the lateral resolution obtained in the numerically processed and up-sampled images. The estimated later resolution, in terms of full-width half maximum (FWHM), was found to be 4.6 μm using a single photoreceptor reflection signal from a magnified image of a photoreceptor mosaic, shown in Figure 5G. The 4.6 μm lateral resolution requires sampling every 2.3 μm to meet the Nyquist criteria but we sampled every 3.3 μm. However, the lateral resolution is improved beyond the Nyquist limit by 8x numerical up-sampling and subpixel image registration of 30 volumes of slightly moving eye. Our approach is similar to the iterative weighted shift-and-add method^51^ and a method that uses a modulation of the phase in the system pupil aperture by deformable mirror (DM).^52^

### Choroidal microstructure

**Figure 6:**
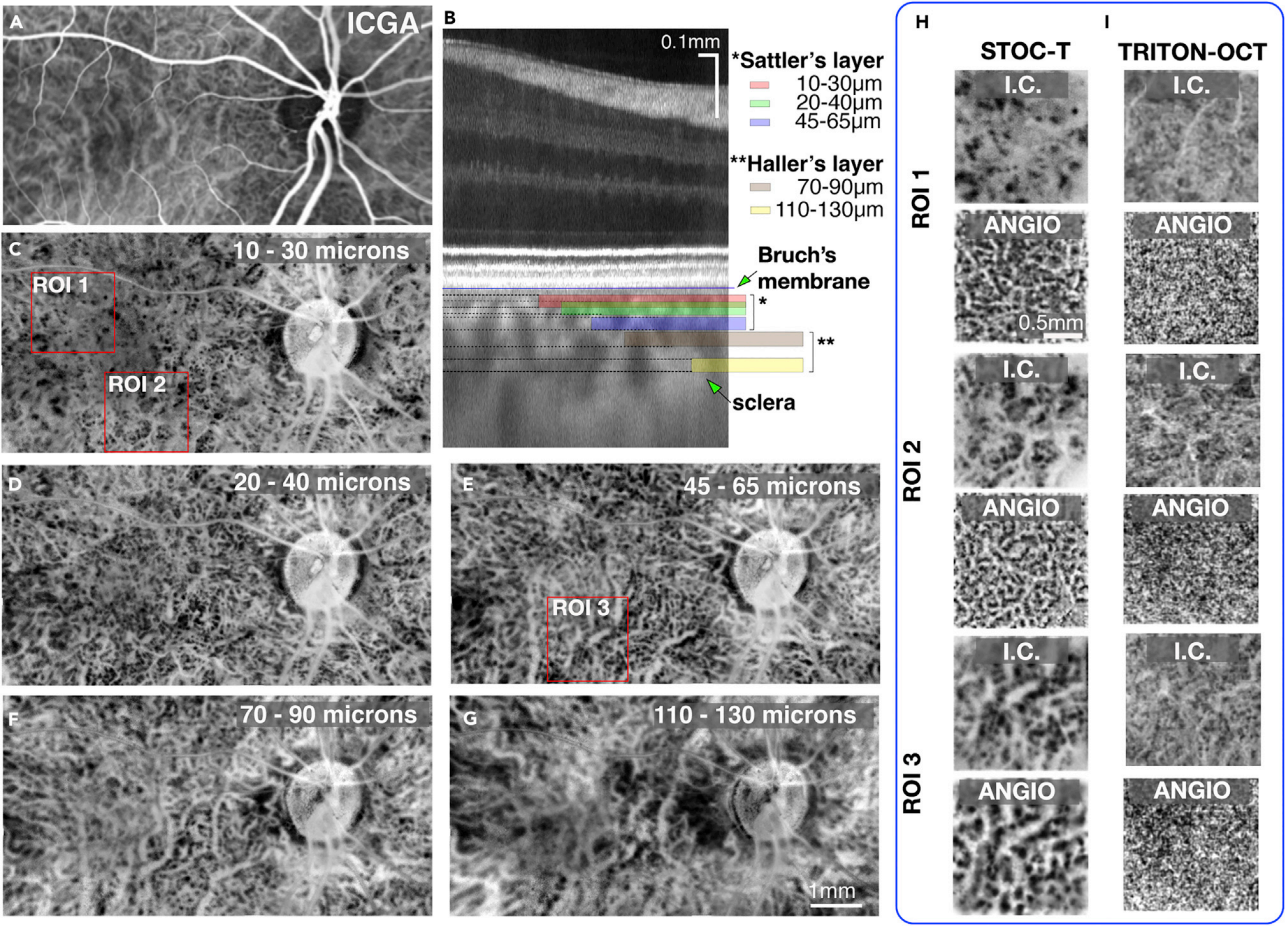
STOC-T Imaging of the choroid – coronal sectioning (A) ICG angiography image exposes mostly the large vessels of Haller’s layer. (B) A representative cross-sectional image with the locations of arbitrarily selected projections from the choroid thickness marked by color bars displayed in panels (C)-(G). (C–G) Contrast-inverted (I.C.) projections derived by averaging STOC-T structural *en face* images (20 μm thick) in the indicated axial range below a line demarcating the RPE and Bruch’s membrane. The images in the range of 10 μm–70 μm represent the Sattler’s layer, and below that the Haller’s layer. Vessel diameter can be seen increasing with the imaging depth. (H) Zoomed-in ROI I - ROI III areas marked with red boxes in panels (C) and (E); corresponding STOC-T angiography reconstructions are shown in the adjacent panels, revealing more details; I.C.-contrast inverted projections. (I) corresponding choroidal Images acquired with swept-source OCT (Triton, Topcon) at the depth of 20 μm and 55 μm for inverted contrast and angio-OCT reconstructions.

The choroid is traditionally divided into three parts at increasing depths: the choriocapillaris, Sattler’s layer, and Haller’s layer. Haller’s layer contains the larger vessels of the choroid, whereas Sattler’s layer contains medium-sized vessels. There is no clear boundary between the two layers and they display significant individual variability.^21^ The thickness of these layers varies significantly between individuals and also within the retina; typically, the choroid is thicker in the subfoveal zone whereas it thins considerably in the optic disc region. As shown in Figure 6B, the complexity of choroidal tissue makes it optically heterogeneous and difficult to image at different depths. ICG angiography images mainly display the large vessels of Haller’s layer, as shown in Figure 6A. In addition, ICG angiography images acquired at different points in time did not reveal choroidal microstructures (Figure S12). The vascular structure of the Sattler’s layers is only visible in the area of the optic disc. By using STOC-T, we can appreciate the richness and complexity of the choroidal vascular system acquired at various depths in the choroid, as shown in Figures 6C–6G. Signals with high local dynamics related to blood vessels are clearly hypo-reflective relative to the static part of the choroid that contains connective tissue, neurons and pigments. To emphasize the vascular structure, we inverted the contrast of the projection images so that the hypo-reflective vessels became hyper-reflective and the vascular architecture became clearly visible, as shown in Figures 6C–6G. There is a noticeable difference in the choroidal structure already with a 10 μm change in depth, as shown in Figures 6C and 6D and 6H, indicating that the images are not a result of projections from the upper layers.

The same effect of varying vascular patterns for different depths is observed for angiography images acquired with STOC-T. In our work, we chose arbitrary choroidal layer thicknesses and their locations deep within the tissue to show the most representative and non-redundant structures (Figure 6B). STOC-T images show a large variation in vascular patterns as a function of depth. To compare the performance of STOC-T versus scanning OCT techniques, we used an identical analysis of images obtained with Triton OCT, allowing reconstruction of structural inversion images, and compared them to the results from STOC-T (Figure 6I). With the scanning OCT instrument, the structural inversion images had significantly lower contrast than the STOC-T images. Furthermore, no correlation could be observed between inversion images and angio-OCT images. Although for STOC- T, these two types of data correlated very well, revealing increased contrast in STOC-T angiography and visualizing the vascular system of different layers of the choroid, as illustrated in Figure 6H for selected regions of interest (ROIs). Therefore, STOC-T angiography images were selected to perform further numerical analyses on various morphometric parameters. It is important to note that both structure (morphology) and STOC-T angiography are derived from the same set of data with no modification of the measurement protocol required.

### Choriocapillaris

**Figure 7:**
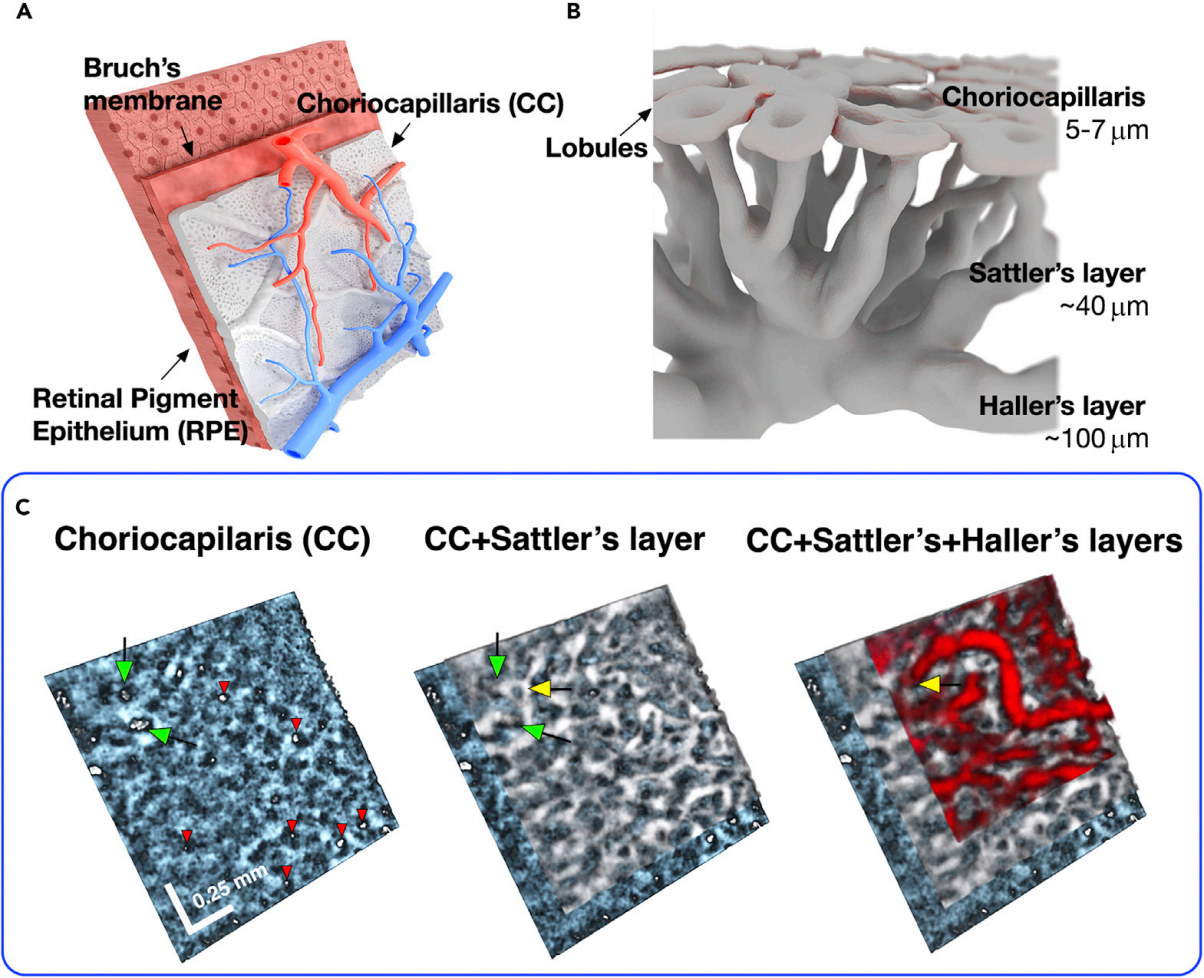
STOC-T Imaging of the choroid in 3-D (A) Drawings showing the blood circulation system in the choroid supplying the choriocapillaris, relative to the localization of the retinal pigment epithelium, as seen from the sclera. (B) Artistic representation of the choroidal layers and choriocapillaris with relative thicknesses of each layer. The diagram shows the lobular arrangement of the choriocapillaris and the increasing size of the choroidal vessels with depth. (C) STOC-T imaging of the choroid reveals inter-space relationships among the retinal vascular layers, rendering three distinct choroidal layers. White bars represent 0.25 mm; Green arrows point at two exemplary locations of Sattler’s layer vessels inserting into the plane of the choriocapillaries, approximately perpendicularly to the CC. The white openings correspond to insertions of respective feeding and draining Sattler’s vessels into the outer surface of the capillaries (other exemplary locations are marked with red arrows), and indicate the functional lobular architecture. In contrast, the large vessels of Haller’s layer heading through the medium vessels towards the openings are marked in yellow. Data were rendered in FluoRender free software (http://www.sci.utah.edu/software/fluorender.html).

The choriocapillaris (CC) is responsible for providing oxygen and nutrients to the outer retina, as well as removing the metabolic waste from the RPE and photoreceptors. The CC is a dense vascular monolayer of interconnecting capillaries adjacent to the outer surface of the Bruch’s membrane and the RPE cells, as illustrated in Figure 7A. The thickness of the CC layer ranges from 6.8 μm in the *fovea centralis* to 5 μm 1 mm away from the macula.^33^ Together with Bruch’s Membrane (BM), which has a distinct internal structure only in visible OCT imaging,^53^ the CC + BM complex is approximately 12 μm thick and is hyporeflective in OCT cross-sectional images, creating a characteristic demarcation line between the bright layers of the pigment epithelium and the choroidal layers. Histological studies have shown that CC vessels have a different morphological arrangement in different regions, forming a dense honeycomb network of freely connected capillaries separated by septa in the macular region, and a polygonal lobular network (as shown in Figure 7B) in the equatorial and peripapillary regions. Although the ability to quantitatively image the CC *in vivo* would be of great value, a clinically useful method that reliably provides this information has not been demonstrated to date.

**Figure 8:**
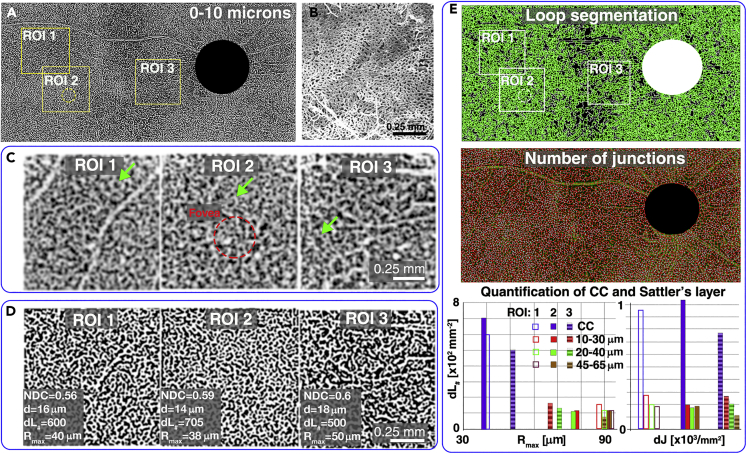
Imaging of the capillary lamina of the choroid (choriocapillaris) and its morphometry (A) STOC-T angiography map (binarized after Frangi Hessian filtering) of the 10 μm-thick layer located next to the RPE, showing the capillary network image; the ROI areas 1–3 represent individual tiles of the mosaic. (B) SEM image showing the morphology of the choriocapillaris (adapted from^54^). (C) Magnified sections of the STOC-T angiography maps (non-binarized version) from the areas highlighted in panel a.; green arrows indicate a small vascular sub-system with a specific morphology recurring in each ROI; ROI 2 corresponds to the area located directly in the Foveal zone as indicated with a red dashed circle. (D) Contrast enhanced STOC-T angiography images (binarized) obtained by using the Frangi-Hessian algorithm. (E) Quantitative analysis of the choriocapillary vascular system by automatic detection of loops and junctions; the graphs show the results of counting loops and density of junctions dJ for the choriocapillaris and three projections from Sattler’s layer, reproduced in Figure 6; NDC - normalized capillary density, d – mean capillary size, dL_#_ - local capillary density, R_max_ – maximum radius of loop present in ROI; a significant difference in R_max_ was observed between the area of the fovea centralis and the area near the optic nerve disc.

Figure 7C shows 3-D rendering of STOC-T data from the choroid area. To better visualize the interrelationship among the 3 different choroid layers, the CC, Sattler’s layer, and Haller’s layer were highlighted with different colors. This colorized depiction demonstrates that with growing depth the diameter of the vessels increases and their density and degree of branching decreases. Green arrows point at two exemplary locations of Sattler’s layer vessels inserting approximately perpendicularly into the plane of the CC. The white openings correspond to insertions of respective feeding and draining Sattler’s vessels into the outer surface of the capillaries (other exemplary locations are marked with red arrows), and indicate the functional lobular architecture. In contrast, the large vessels of Haller’s layer heading through the medium vessels towards the openings are marked in yellow. Figure 8 shows the results of choriocapillaris layer extraction. CC visualization was carried out by using the angio algorithm on 10 μm thick layers.

To produce angiography images, the same data sets were used as in previous sections that allowed image stitching to the size of 9mm×4.6mm. Each 1.7mm×1.7mm image that constituted the montage was analyzed separately with the dedicated STOC-T angiography algorithm. The STOC-T angiography signal coming from the choriocapillaris layer is a relatively weak signal that is detected against a high background signal coming from a highly reflecting structure. It, therefore, required additional adaptive histogram equalization followed by the blood vessel contrast enhancement using Frangi Hessian filtering, as shown in Figure 8D. The ROI areas 1–3 represent individual tiles of the mosaic shown in Figure 8A. To improve visualization of the large number of details in panel a, an image of the mosaic after Frangi Hessian filtering is shown. ROI 2 corresponds to the area located directly in the Fovea Centralis zone as indicated with a red dashed circle. To compare the morphology of the reconstructed CC layer we show an example of a Scanning Electron Microscope (SEM) (adapted from^54^) on a scale identical to the ROI1-3 in Figure 8B. It can be seen that the dense vascular network is reproduced in the STOC-T image with similar morphological features. In addition, the functional lobular nature of the choriocapillaris structure is manifested by the presence of small vascular subsystems of specific structure (indicated by green arrows in Figure 8C), whose recurrence suggests their function of supplying or draining blood from the choriocapillaris, shown in Figure 7A.

### Quantification of choriocapillaris morphometry

STOC-T enables quantitative analysis of the choroidal layers to identify an effective biomarker for assessing disease progression or treatment effects. Because of the specific reticular shape of the choriocapillaris and Sattler’s layers, we proposed a morphological analysis based on two easily identifiable and robust imaging parameters, the morphology of the loops surrounding the no-flow zones and the number of connections between the vessels. The number of loops per 1mm^2^ corresponds to the density of the vascular network and was named here as a local capillary density, dL_#_. In turn, the size range of the loops indicated the morphological variability of the analyzed vascular area. To characterize this variability, we identified the maximum loop size present in a given area in a significant number of segmented loops; we call this value the maximum loop radius R_max_. The R_max_ value was obtained automatically by counting the area of the region surrounded by the loop in the binarized image using ImageJ. Such analysis was implemented for each *en face* cross-section of interest. From all the calculated area values, the maximum values were selected and from them the radius value was calculated assuming that each "island" can be approximated to have a circular shape. The results were collected in a graph (Figure 8E), which showed a large disparity between the vessel density in the CC layer relative to the first of the arbitrarily selected depths of the Sattler’s layer (a 20 μm slab with a center located 20 μm away from the RPE). This analysis revealed an approximately 5-fold higher capillary density in the choriocapillary layer at a distance of planes only 10 μm apart in depth. The R_max_ value also showed specificity for different ROI locations for the choriocapillaris. According to anatomical knowledge,^54^ the density of capillary vascular plexuses is the highest and least variable in the area under the fovea centralis; whereas in the peripheral parts of the retina the system thickens, creating a distinct lobular pattern. Assuming that normalized capillary density NDC^31^ is constant (0.6 for all ROIs) the mean value of the loop radius corresponds to the mean value of the capillary size, *d*. Another way to characterize the morphology of the vascular system is to count all the vascular junctions present in a given area. Figure 8E shows the result of counting the density of junctions (dJ) in the choriocapillaris layer and in corresponding areas of different depths of the Sattler layer for ROI1-3. The values of dJ in the dense choriocapillaris structure change similarly to the number of loops reaching maximum values in the macular area and lower values in the area close to the optic nerve disc. The change in the connection density is less significant at other depths in Sattler’s layer.

## Discussion

The results of this study demonstrate that STOC-T is capable of *in vivo* visualization of major retinal and choroidal layers, including that of the choriocapillaris. Scanning OCT has not yet been able to obtain detailed images of the morphological structure of the retina and choroid from a single data set without adjusting the scanning protocol or using sophisticated hardware solutions such as AO-OCT. In contrast, STOC-T enables imaging of the choriocapillaris, choroid, and details of the ILM, NFL, or photoreceptor mosaics with a fairly simple system. *In vivo* imaging of the choriocapillaris layers is very challenging for currently existing techniques, because of the small thickness of the choriocapillaris layer, the strong extinction of the optical signal, and the rapid leakage of dye from the fenestration of the capillaries themselves. Consequently, most studies of choriocapillaris function and morphology related to retinal disease etiology and progression have been limited to postmortem histology or indirect observations. The only method that indicates the possibility of *in vivo* imaging of the choriocapillaris is currently OCT. However, the choriocapillaris reconstructions achieved by OCT are too low in contrast to be analyzed quantitatively, as evidenced by the lack of results showing variation in choriocapillaris density for different locations in the posterior pole of the eye.^55^ The low contrast of OCT reconstructions is because of the presence of speckle noise and the limited lateral resolution of current commercial OCT systems. Also, the depth of the typically analyzed layers is more indicative of one of the first Sattler’s layers than of the choriocapillaris itself.^56^ STOC-T provides choroidal images with better image quality compared to scanning OCT, which is somewhat counterintuitive, because scanning OCT uses confocal detection in addition to the temporal gating, which should in principle allow deeper imaging because of better suppression of multiply scattered light. However, as we have shown in this work, almost completely decoupling of lateral resolution and imaging depth is possible in STOC-T, which establishes a more optimal operating condition for chorioretinal imaging. The decoupling is achieved because the imaging depth in STOC-T is not limited by the illuminating beam (or NA) to the same degree as in confocal scanning OCT. For example, for the effective lateral resolution of 4.6 μm that we obtained in STOC-T retinal imaging, we could image a 500 μm thick retina, as well as a 150 μm thick choroid. DOF of 650 μm is also achievable with the Triton OCT system at 1050 nm; however, the difference is that the Triton OCT has a lateral resolution of 20 μm, which is 4 times poorer than the STOC-T. A corresponding scanning OCT system achieving 5 μm lateral resolution would have a limitation in imaging depth (related to the Rayleigh parameter) of about 50 μm, which would fundamentally prevent simultaneous measurement of the retina and choroid. STOC-T also acquires higher quality images through temporal and spatial coherence gating because the speckle size is as important as the resolution in retinal images. As such, the advantage of STOC-T is not necessarily the achievable imaging depth; rather, the key advantage comes from the nearly three-times smaller speckle size (3.3 μm) compared to scanning OCT.^40^ Accordingly, the speckles in STOC-T do not mask the real retinal/choroidal structures to the same degree as do the larger speckles in scanning OCT, which is limited to ∼10 μm resolution. In addition, STOC-T can also reduce the speckle contrast^40^ by averaging multiple volumes. The speckle size reduction can be explained by the fact that the speckle size in wide-field imaging systems is largely determined by the pupil size and is insensitive to optical aberrations.^57^ This is in contrast to scanning OCT, where aberrations force a limit in pupil size (to around 2–3 mm) over which the backscattered light from the retina can be collected through the confocal pinhole (e.g., fiber core).^58^ In FD-FF-OCT the signal can be collected over the whole pupil diameter (of 6–8 mm) that could therefore reduce the speckle size by a factor of 3–4 compared to a scanning OCT. Although the speckle size in wide-field imaging (such as FD-FF-OCT/STOC-T) is independent of ocular aberrations, the resolution is dependent. Fortunately, both the higher order aberrations and the defocus can be taken care of by computational optical aberration correction that is possible in FD-FF-OCT because of inherent phase stability.^59^^,^^60^ Such correction, for example, enables generating sharper images of photoreceptors,^37^ a property which is retained even when the phase of the laser has been scrambled.^46^ Such improved resolution is much more difficult to achieve in scanning OCT because of phase stability issues arising from the scanning jitter and object motion.^61^ In addition, for strong defocus in scanning OCT, the SNR decreases dramatically making numerical correction even more difficult. The STOC-T system described here is not fully incoherent because only a small part (115 μm) of the pupil plane is filled with the illumination beam. However, that is enough to remove most of the visual crosstalk from the images. The remaining crosstalk could be removed by creating smaller coherent areas on the sample, which could be achieved by employing a multimode fiber with larger than 50 μm core (given the same numerical aperture and wavelength). However, that would require longer fiber (to separate all the travelling modes),^62^ which would automatically result in higher light losses in the fiber because of scattering and absorption within. Another important parameter differentiating STOC-T from scanning OCT is the speed and scanning(less) mechanism. Namely, STOC-T is able to acquire 3D volumes of the retina in milliseconds because each pixel of the ultrahigh-speed camera acts as a separate single-point detector, enabling simultaneous signal acquisition from adjacent retinal points. This parallelization makes mechanical scanning obsolete and, subsequently, makes volumetric signal detection faster compared to the scanning OCT that employs a single-point detector and galvanometer scanners. The volumes are normally averaged to increase the image contrast (SNR), but the effective A-scan is still very high; namely, 3 MHz when 10 volumes are averaged. Such images are also devoid of point-scanning artifacts encountered in scanning OCT. STOC-T can computationally recover aberrated signal and restore resolution. Thus, for example, details of the photoreceptor layer become visible, which is possible despite phase randomization^46^ with the multimode fiber.^45^ STOC-T also utilizes available photon budget more efficiently, because aberrated signal is normally rejected by the pinhole in the scanning OCT, whereas in STOC-T it is detected and can contribute to the signal and also reduce speckle size. Furthermore, implemented real-time axial imaging of the retina was instrumental here in producing large (montaged) images of the retina and choroid, as it enabled finding the focus quickly each time a new imaging location had to be imaged. To summarize, in this work we have documented many advantages of the STOC-T technique compared to regular scanning OCT.

(a) STOC-T has longer imaging range (when both systems have the same lateral resolution) because of the differences in the illumination and the ability in STOC-T to computationally correct for defocus (and other aberrations).
(b) STOC-T can achieve higher lateral resolution when imaging human retina because larger pupil aperture of the human eye can be used to detect the backscattered signal from the retina.
(c) STOC-T can image the choriocapillaris layer and do it over a larger axial and lateral extent compared to hardware-based AO-OCT.
(d) Images of the choriocapillaris acquired with STOC-T can be used to derive quantitative morphometry metrics.

In conclusion, we have advanced STOC-T technology with a series of developments that help us to generate high quality retinal and choroidal images with a simple and robust optical layout, and make it more suitable to use in a clinical environment. To demonstrate the benefit of STOC-T we have demonstrated large FOV imaging of the choriocapillaris.

### Limitations of the study

The main limitation of the proposed technology at this time is the current price of the camera, which is around 100,000 euros. However, it is reasonable to expect that the price will decrease with an increase in the production volume of the cameras. The absence of confocal gating because of the use of a camera for signal detection that mechanically rejects out-of-focus light could be seen as another disadvantage; however, for chorioretinal imaging with STOC-T, we do not see a compromise in the imaging depth as one might expect. Indeed, we can image all of the major layers and, moreover, we can visualize hard-to-image layers over a large transverse and axial extent. Currently, the data can only be analyzed offline because of the low transfer rate between the camera and computer. It also requires substantial computer power to process all of the vast amount of generated data, which, however, can present itself for analysis with data-hungry algorithms such as deep learning.

## STAR★Methods

### Key resources table

**Table:** 

REAGENT or RESOURCE	SOURCE	IDENTIFIER
**Software and algorithms**		

Matlab	Mathworks Inc.	2019b
LabView	National Instruments	2017
Python	Python Software Foundation	3.9
FluoRender	http://www.sci.utah.edu/software/fluorender.html	2.23.1
AngioTool	http://angiotool.nci.nih.gov	5.0
Mendeley Data	https://data.mendeley.com/datasets/9pzm3mbp7w/1	https://doi.org/10.17632/9pzm3mbp7w.1

**Other**		

Swept source laser	Superlum	BS-840-2-HP
Multimode fiber	Thorlabs	FG050LGA, 300 m length
Ultrafast camera	Photron	SA-Z 16GB
Preview camera	Alkeria	NECTA N4K-7-F
Rod mirror	Edmund Optics	#1mm Diameter 45° Rod Mirror Aluminum Coated, 47–628
Microscope objective	Olympus	PLN10X

### Resource availability

#### Lead contact

Further information and requests for resources and should be directed to and will be fulfilled by the lead contact, Maciej Wojtkowski (mwojtkowski@ichf.edu.pl).

#### Materials availability

No materials, new unique reagents were created as a result of this work.

### Experimental model and subject details

The study was conducted at the International Center for Translational Eye Research at the Institute of Physical Chemistry, Polish Academy of Sciences. The study protocol was approved by the ethics committee at the Collegium Medicum of Nicolaus Copernicus University (KB 311/2018). Written informed consent was obtained from a healthy, 45-year-old man who participated in the study. The volunteer was measured without the use of additional pharmacological agents. Measurements were taken in a sitting position with the forehead resting on the chin rest without darkening the room.

### Method details

#### STOC-T system

Figure S1 shows a simplified STOC-T system. The system employed a swept laser source (Broadsweeper BS-840-2-HP, Superlum), tunable in the range of 800–878 nm with the instantaneous linewidth of ∼0.1 nm and the output power of 25 mW. The laser light was coupled into the 300 meters-long step-index multimode fiber (Thorlabs, FG050LGA) with 50 μm core (NA = 0.22) for reducing spatial coherence^62^ and delivered to the imaging system.

The light emerging from the multimode fiber was first sent through the modified illumination arm that included a small rod mirror and a line camera, which were used to implement a real-time axial retina preview without compromising the overall performance.^62^ A 50/50 beamsplitter (BS) in the interferometer reflected half of the light (5 mW) towards the eye and the remaining to the reference arm, which attenuated the light to ∼10% in the double path. The tip of the fiber was effectively magnified 2.3-times in the pupil plane of the eye, to 114 μm. The backscattered light from the retina and light reflected from the reference were recombined by the beamsplitter and imaged on the ultrahigh speed camera (Fastcam SA-Z, Photron) through one of the BS facets and onto the line camera (Alkeria, Necta N4K-7-F) through another BS facet (the same that the sample is illuminated through). The lens before the eye in the sample arm was used for adjusting the focus of the retina on the camera by translating it along the optical axis, which enabled accounting for the refraction error in the eye. Images were acquired at 60 kHz with the camera while the swept source was continuously tuned at 8700 nm/s. In total 512 images were acquired per volume, which resulted in a 0.15 nm step between the recorded images and led to the volume acquisition time of ∼8.6 msec. Such illumination formed by the multimode fiber created a large enough DOF to enable volumetric acquisition of the entire thickness of the retina. To measure DOF, a mirror was imaged in the sample arm by employing an identical microscope objective, like in the reference arm, resulting in the sample imaging FOV of 2.46 mm × 2.46 mm. A translation stage was stepped along the optical axis in the sample arm and an interference image was recorded at each axial position at fixed wavelength. The interference images were then used to derive the DOF curve, shown in Figure 2A, by estimating the fringe contrast for each image. The DOF measurement was estimated to be at least 2.9 mm (FWHM), but the measurement was limited by the temporal coherence length of the laser. For refocusing experiments with the USAF target, volumetric data were acquired, and so sampling (number of images acquired per single laser sweep) also had an effect on the achievable imaging/refocusing range. Nevertheless, the coherence length limitation is more fundamental here than sampling, as the coherence length is fixed to the estimated value of 3.18 mm, which can be calculated from $0.44\lambda^{2}/\delta \lambda$, where δλ=0.1nm is the instantaneous linewidth of the laser that cannot be changed, and λ is the central wavelength (850 nm). The sampling, however, can be easily changed by varying the number of images acquired per laser sweep. However, number of sampling points (images) are preferred to be kept at the minimum in order to keep the acquisition time to the minimum. The sampling in OCT imaging mode defines the imaging depth range (roll-off) that can be estimated from $N\lambda^{2}/4\Delta \lambda$ as 1.2 mm (which should be counted from the 0 mm position in Figure 2), where Δλ is the tuning range of the laser (78 nm) and N is the number of images acquired per one sweep (512 in this work). Since ∼250 spatially independent modes are excited in the multimode fiber, the same number of independent spatially coherent areas will be formed across the end of the fiber tip, and eventually on the sample, as this number cannot be changed by any lens transformation.^43^ Therefore, the diameter of the coherence areas can be simply estimated as FOV/√N, which for imaging the USAF target can be calculated to be larger than $\frac{2.46mm}{\sqrt{250}}=150\mu m$ (because some of the coherent areas will be outside of the FOV). The Rayleigh range of the coherence area can then be estimated to be around 80 mm by using the $\pi \Delta x^{2}/\lambda$ relationship for a coherent laser beam. The axial resolution was measured to be 5 μm, which is close to its theoretical value of 4.1 μm, as calculated from $0.44\lambda^{2}/\Delta \lambda$, with the discrepancy arising due to the formula’s assumption that the spectrum of the source has a Gaussian distribution (which it does not). The lateral resolution (in the human retina) was estimated to be 4.6 μm obtained after up-sampling and defocus correction. The imaging sensitivity for the un-averaged reconstructions was 72dB and reached 91dB after averaging 30 volumes. We estimated that the field of view on the retina (for one volume) was 1.7 mm × 1.7 mm for 512 × 512 images.

#### *In vivo* measurement procedure

A 3D translation stage-mounted chinrest was used to align the head of an individual for the measurement. Real-time axial images of the retina provided by the line camera were used as a visual guidance to (I) find signal from the retina by axially translating the chin rest until optical paths between the sample and reference arms were matched; and (II) to optimize the signal in terms of axial image sharpness by adjusting a lens before the eye, which allowed us to account for the refraction error in the eye. Once the desired eye starting position was found, multiple volumes were acquired at multiple retinal locations in order to image a large part of the retina with high SNR. Between each retinal location the volunteer was asked to focus on a different part of a ‘ship game’ board, while keeping the head position fixed. The acquisition interval between two consecutive positions was about 20 s, which gave a total measurement time of about 10 min.

#### Eye safety

To determine the risks of using a laser light source for STOC-T experiments, we used the ANSI Z136.1–2014 standards and detailed instructions found in the following publications:^63^^,^^64^ Because of the potential risk to the anterior (cornea and iris) and posterior chamber of the eye (retina), such a method was used to indicate the greatest risk by analyzing the worst-case scenarios for each of these anatomical parts separately.

The Broadsweeper emits CW radiation tuned over the spectral range of 800–870 nm, which was coupled into the step-index MM fiber (Ø50 μm core, FG050LGA, Thorlabs). The light from the MM fiber was collimated by lens f = 11 mm; subsequently, lens f = 50 mm imaged the tip of the MM fiber onto a small (1 mm diameter) 45°rod mirror. The mirror reflected all of the illumination towards an interferometer, since the image from the fiber core that formed on the surface of the mirror was magnified (4.5×) to approximately 227 μm. Lenses f = 100 mm and f = 50 mm reimaged the mirror, as well as the fiber tip, in front of the human eye to around 100 μm of diameter. The image of the fiber tip is a circular extended secondary source, located in the focal plane of the eye, which results in a collimated beam with diameter of 1.7mm on the retina. Considering the Standard Gulstrand eye model, the focal plane is located at a distance of f = 17 mm from the secondary principal plane, which in turn is located at a distance of about 1.6 mm from the corneal surface. This means that the macula with the highest power density is located at a physical distance of about 15 mm from the corneal surface. In this configuration, the beam size on the cornea and iris are about 1.5 mm. It should be noted that a necessary requirement of the STOC-T configuration is that the beams must be collimated in both arms of the interferometer. Without this condition, it is not possible to obtain an interferometric signal and thus a volumetric reconstruction. This means that obtaining a signal in the preview is only possible in the configuration when the beam is focused in front (at least 1cm) of the volunteer’s cornea.

Depending on the state of accommodation, refractive error and also individual differences in the shape of the eyeball, the location of the spot with the highest optical density may change and, in the worst case, may coincide with the cornea. However, in the case of the volunteer presented in this paper, it was measured that there was a distance of about 2 cm between the image of the multimode fiber and the corneal front. In addition, in the process of positioning the STOC-T device relative to the patient’s/volunteer’s head, the position of the point of highest power density may overlap with the position of the iris or cornea. During the measurement, the patient’s head rests on the chinrest with fixation based on the visual stimulus without additional ties to stabilize the head or eye. During positioning, the patient/volunteer can move the eye and blink. The head typically makes involuntary movements on a scale of hundreds of micrometers during the few minutes required for positioning. During this time, the collimated beam falls on the retina and the patient/volunteer can stabilize the eye position by observing the collimated infrared beam. At the start of the measurement, the operator asks the volunteer/patient to open the eye and not blink for less than 300ms in which a series of 30 STOC-T volume measurements are taken. In such a setup, we assume that the retina is illuminated continuously for more than 10 s (during patient’s adjustment). Due to the relatively small size of the focused spot in three dimensions, involuntary movements of the patient, blinking and movements of the device during head positioning make it unlikely to illuminate one position of the iris or cornea during patient positioning. The basic safety analysis scenario assumes that for retinal illumination we will refer to the Maximum Permissible Radiant exposure at the cornea for Maxwellian view. For the retina, cornea and iris, we assume the worst-case scenario when the eye is exposed for more than 10 s. For the retina, we assume extended source conditions while for the cornea and iris we assume that the risk source is a beam focused directly on one of these two organs.

As a first step, we point out the recommendations for corneal and iris safety (paragraph 8.3.4 of ANSI Standard Z136.1–2014). According to these recommendations, irradiation of large areas of the retina in the 400nm to 1400nm spectral range (i.e., the "Maxwell view") can cause high irradiation of the anterior segment of the eye - particularly the cornea and highly pigmented iris. For the cornea and crystalline lens within 1 mm of the aperture (area of Scornea = 7.85 × 10–3 cm2) irradiance shall not exceed the value of 25 t-0.75 W cm-2 for t < 10 s and 4 W cm-2 for t > 10 s. The exposure of the iris must not exceed five times the MPE of the skin (see paragraph 8.4 and Table 7 of ANSI Standard Z136.1-2014). According to the ANSI standard guidelines, MPEs are expressed (normalized) relative to the area of the limiting aperture and therefore the limiting aperture should be used for measurements or calculations with all MPEs. The limiting aperture is the maximum circular area over which irradiance or variable exposure should be averaged (paragraph 8, page 63). For iris hazard analysis, the limiting aperture for corneal exposure for all wavelengths is used for wavelengths from 1200 to 1400 nm, i.e., an aperture of 3.5mm in diameter (Siris = 9.62 × 10-2 cm2). In the case of the retina, we assumed that we were dealing with illumination by extended source of angular size F = 0.1 rad (calculated with assumption that the focal length of Gullstrand eye is feye = 17mm).

Recalling the terminology from the publication, F. C. Delori, R. H. Webb, and D. H. Sliney, "Maximum permissible exposures for ocular safety (ANSI 2000), with emphasis on ophthalmic devices," J. Opt. Soc. Am. A 24(5), 1250–1265 (2007)) we determine MPHc – Maximum permissible radiant exposure at the cornea [J/cm2], which for times longer than 10s may be expressed in [W/cm2]. In addition, following the same publication, we choose to express the exposure limits for ophthalmic applications in intrapupillary radiant power MP F [Watts], that corresponds to the quantity measured by power meters. For retinal hazard estimation the maximum permissible radiant power is expressed:

$$MP{\text{Φ}}_{ret}=MPH_{C}*\left[\frac{S_{Retina}}{P}\right],$$

where: S_Retina_ - the area of a 0.7 cm diameter pupil = 0.385 cm^2^, P – pupil factor = 1.

In the case of Iris and Cornea we calculated maximum permissible radiant power as:

$$P{\text{Φ}}_{Cornea.iris}=MPH_{Co}*S_{Cornea/iris},$$

where S_cornea.iris_ – limiting apertures used for measurement of exposure (Table 8a,b of ANSI Standard Z136.1–2014). MPH values likewise threshold exposures for thermal damage to ocular tissue vary linearly with the diameter of the illuminating spot.^64^ Therefore, in the absence of strict indications for a various sizes of focused beam illuminating the cornea and iris, we can estimate MP F 0.1 for the worst possible case of illuminating the cornea and iris with focused light by dividing the MPHco/iris values by the ratio of the measured macular diameters and the diameters of the limiting apertures.

Detailed calculations of maximum permissible radiant exposures MPHc and maximum permissible radiant powers MPΦ are presented in Table S1.

Concluding: 1. Due to the use of an extended light source, the tested value of light power illuminating the eye is more than 4 times lower than the value of maximum permissible power and poses no danger to the retina;

2. In the STOC-T measurement configuration, the human eye acts as a beam collimator, so the 100 μm image of the 50 μm core of the multimode optical fiber is at a distance of about 1.5 cm from the corneal surface, resulting in a beam diameter of about 1.5 mm illuminating the cornea and iris. In such a configuration, the power density illuminating these organs is 250 mW/cm^2^, which results in 16- and 6-times lower power densities, respectively, than the limitations associated with safety standards;
3. In the worst possible case, when the patient’s eye is in the process of alignment, it may happen that the tip of the multimode optical fiber is imaged directly on the cornea or iris. In such a situation, the recommended power for long illumination t > 10 s are slightly exceeded. However, such a situation is unlikely due to the lack of eye immobilization and the patient’s ability to blink and move the eye freely. In addition, during alignment, the operator moves the manipulators moving the beam until it reaches the optimal measurement position. In such a situation, it should be assumed that the effective time for the focused spot to illuminate one location is less than 1 s. For such assumptions, the power density of the light used in the experiment is four and two times lower than the threshold for acceptable values for the cornea and iris, respectively.

#### Data analysis

We acquired spectral-domain fringe patterns – a series of 2D images for a variable wavelength (X, Yλ) using the setup depicted in Figure S1(a) at the speed of 60 000 fps. The spatial dimensions (X, Y) of each image were set to 512 × 512 pixels. As sketched in Figure S2, we recorded 512 such images ($\lambda_{1},\lambda_{2},\ldots \lambda_{512}$) while tuning the laser wavelength (λ) from 800 nm to 875 nm with the speed of 8700 nm/s. This corresponds to a single volume acquisition time of 8.6 ms with an intervolume delay (idle) time of ∼0.3 ms. This delay is required to reset the configuration of the laser tuning. With those parameters, more than 110 volumes per second could be recorded. However, we typically acquire N = 10–30 volumes per location, resulting in a total acquisition time of ∼90–265 ms.

The mean from each line (along λ,A-scan) was first subtracted, followed by apodization with the Hamming window function, and conversion from spectral to depth domain (λ to z) by Fast Fourier transformation (FFT) with zero padding (Figure S4). The *en face* images of the retina were then spatially filtered (Figure S5) to remove low-frequency speckles arising from the multimode fiber. To estimate the spatial filter, we calculate the 2D FFT of each *en face* plane. By doing so, we get the 3D stack of complex values. We then integrate amplitudes of the resulting stack along Z to obtain the 2D image. This image contains distinguishable circles. As the location of these circles might vary between measurements, we detect them automatically. So, we use the Hough circles transformation preceded by adaptive thresholding. Usually, this process finds several spurious circles. We reject them based on the radius and distance from the center. We use predefined thresholds that only keep rings with radius in range (15–130 pixels) and are located not further than 150 pixels from the center. Once the spatial filter is estimated, we use it to mask selected spatial frequencies and then perform inverse 2D FFT at each depth (Z) as sketched in Figure S5. As a consequence, we suppress fixed pattern noise and low-frequency speckles from the multi-mode fiber. Volumes were then corrected for chromatic dispersion iteratively, which also corrected for any possible axial motion of the eye during the laser sweep (Figure S6). We use an iterative approach with kurtosis as a quality metric and five adjustable parameters. Specifically, we Fourier-transform the complex signal along XZ or YZ (B-scans) and then multiply it by the phase corrector: $\exp\left[i\overset{5}{\underset{i=1}{\sum }}a_{i}{\left(\omega -\omega_{0}\right)}^{i}\right].$. Here, ω=2π/λ denotes the optical frequency, $\omega_{0}$ is the center optical frequency, and $a_{i}$ are adjustable parameters. Then, we compute the inverse Fourier transformation and quantify the sharpness of the resulting B-scans. We continue this process by changing coefficients $a_{1},a_{2},\ldots ,a_{5}$ until the sharpness metric (kurtosis) is maximized (Figure S6). The resulting phase factor is then applied to all B-scans in the volume. Finally, the volumes were corrected for depth-dependent defocus by using a sub-aperture approach (Figure S7).^65^ Higher-order aberrations were then corrected iteratively by optimizing the *en face* (x,y) 4 times up-sampled images (with bicubic interpolation) at selected depths.^46^ The aberrations were parameterized using 21^st^ Zernike polynomials, excluding piston, tilt, and tip (Figure S8). Finally, the processed volumes were aligned, up-sampled with bicubic interpolation, registered using a correlation-based approach and integrated incoherently to increase SNR. The resulting volume was flattened with respect to the photoreceptor layer. To register volumes, first, we selected 20–30 XZ B-scans from two volumes being aligned (reference and moving volume). We then estimated translation between those B-scans using the subpixel registration. Subsequently, we applied the estimated translation to B-scans from the moving volume, and then calculated structural similarity (SSIM) index between reference B-scans and the registered moving B-scans. Afterwards, we chose the translation vector along XZ, $T_{XZ}=\left[t_{X}^{\left(XZ\right)},t_{Z}^{\left(XZ\right)}\right]$ corresponding to B-scans with the SSIM index. We repeated this process for YZ B-scans, which yields translation vector along YZ, $T_{YZ}=\left[t_{Y}^{\left(YZ\right)},t_{Z}^{\left(YZ\right)}\right]$. Given two vectors we constructed the 3D translation vector: $T=\left[t_{X}^{\left(XZ\right)},t_{Y}^{\left(YZ\right)},\frac{1}{2}\left(t_{Z}^{\left(XZ\right)}+t_{Z}^{\left(YZ\right)}\right)\right].$ We then used this vector to translate the moving volume. We repeated this process for other volumes in the dataset. Finally, the amplitudes of the aligned volumes were averaged to achieve the structural image of the eye. The registered volumes, before averaging, were also employed to derive angiography images. STOC-T angiography was obtained by using the Complex Differential Variance method (CDV) applied to the consecutive pairs of complex-valued volumes.^66^ Then the result was convolved with a three-dimensional kernel (Hamming 7x7x7 pixels), and averaged. The *en face* angiographic projections were extracted from averaged volumes by selecting the pixels with intensities above a pre-defined threshold. We have found that the optimal projection angiography maps are obtained by calculation of a mean value map along the Z-axis divided by the maximum value map within the projection range.

The two-dimensional images extracted from processed volumes or angiography volumes were flattened (Figure S9) and stitched together to achieve wide-field images, as depicted in Figures 5 and 6 from the main text. The stitching procedure is divided into six main steps (Figure S10): (1) Image registration; (2) Image warping; (3) Flow signal compensation; (4) Weights map creation; (5) Seamless stitching line detection; (6) Final image blending. To register images we first estimate translation, using phase cross-correlation.^67^ To improve accuracy and robustness of finding the translation, we applied the cross-correlation technique only for the overlapping region of two images. Given an estimate of the displacement between the image pair ($t_{x},\;t_{y}$), we are able to calculate an affine transformation matrix, $A=\left[\begin{array}{lll} 1 & 0 & t_{x}\\ 0 & 1 & t_{y}\\ 0 & 0 & 1 \end{array}\right]$ between the images as: $p_{2}=Ap_{1},\;A$, where $p_{1}$ and $p_{2}$ are matching points in stitched images, shifted image and target image, respectively. Then, we warp shifted images to the target image coordinate system. Ideally overlapping the areas of target and warped images should not reveal a seam at the edge of the composite image, but practically each matching pixel pair exhibits intensity differences because of a number of uncontrolled effects (vignetting, motion, registration errors, *etc*.).

To homogenize stitched images, we performed four post-processing steps: flow signal compensation^68^; weights map creation; detecting the stitching line in low-perception areas; and a seamless multi-band blending algorithm.^69^ To get rid of the blurring effects and improve quality of the stitching images we adopted a strategy for finding the optimal seam lines in the overlapping area to bypass obvious dislocations and discontinuous areas. To do so, we applied a simplified seamless stitching method based on a human visual discrimination and attention algorithm.^70^ The main idea of this algorithm is to quantify visual perception of the overlapped region of images to indicate the influence on the stitching result. The visual perception can be expressed as a weights map (WM) that is a combination of four processing steps: visual non-linearity (NL); luminance difference (LD); visual masking property (M); and visual saliency maps (V). These steps are formulated as: $WM=\alpha_{1}\;NL+\alpha_{2}LD+\alpha_{3}M+\alpha_{4}V$, where $\alpha_{1}-\alpha_{4}$ are adjustable parameters. An optimal stitching line is then detected based on a weight map that indicates the pixel priority route. We applied a dynamic programming to calculate the lowest energy path from the top of the image to the bottom. Such a selected stitching line allows us to avoid discontinuous edges that strongly attract the attention of the human eyes. Finally, images are stitched at the selected stitching line by multi-band blending^69^ to produce a seamless mosaic image. Based on our experimental data we observed that the quality of stitching images is strongly dependent on the fraction ($\alpha_{1}-\alpha_{4}$) of particular visual perception factors in the final weights map, and can vary for different layer depths of the retina. Thus, we use different values of parameters $\alpha_{1}-\alpha_{4}$ for particular ranges of retinal layers.

Finally, for quantitative analysis, the *en face* angiographic images were enhanced through adaptive histogram equalization, followed by blood vessel contrast enhancement using Frangi-Hessian filtering with sigma = 1pixel. The image was then binarized using the Phansalkar method with a local window radius of 15 pixels.^71^ Axially flattened binarized maps were processed to identify local vascular cross-sections and calculate local maxima, and were then masked for skeletonizing.^24^ Loop analysis allowed us to indicate the range of capillary sizes and density for different locations, both laterally and vertically, for the CC and Sattler’s layers at different depths. In addition, spatial parameters related to capillary branching, such as total number of junctions and density of junctions in vessels, are performed using a semi-automated computational tool.^72^

#### Determination of DOF on reflecting phantom

Determination of lateral resolution was performed using an “artificial eye” comprising a 10× objective (NA = 0.25) and USAF test target. The purpose of this analysis was to obtain information on the relative changes in resolution and in image intensity over a 1-mm range. Images of USAF Test Target surface were obtained from STOC-T measurements by averaging 20 en-face reconstructions. 5 OCT reconstructions of the USAF Test Target obtained for 5 different positions of the target relative to the 10x lens causing severe defocus were analyzed. The defocus correction algorithms described in this paper were then applied. Comparative measurements with the scanning OCT method were obtained using an in-house-made set-up with parameters corresponding to the STOC-T system. In particular, care was taken to match the numerical apertures and effective lateral resolution in both systems. Again, the USAF test target was moved relative to the 10x lens, compensating for the position of the reference mirror.

## Data Availability

• Raw data from STOC-T measurements (2sets of 32 separate files of 512x512x512 pixels, binary 16-bit integer, little-endian.mraw) that support the findings of this study have been deposited Mendelay Data: https://data.mendeley.com/datasets/9pzm3mbp7w/1 (https://doi.org/10.17632/9pzm3mbp7w.1). • Matlab/LabView codes are available from the lead contact upon request. • Any additional data supporting findings on this study are available from the lead contact upon request (mwojtkowski@ichf.edu.pl).
