# Supplementary material for: Spatio-temporal optical coherence tomography provides full thickness imaging of the chorioretinal complex
Source: iScience. 2022 Nov 5;25(12):105513. doi: 10.1016/j.isci.2022.105513 (PMC9676634; doi:10.1016/j.isci.2022.105513)
Supplement: Document S1. Figures S1–S12 and Table S1 [file mmc1.pdf]

iScience, Volume *25*

Supplemental information

Spatio-temporal optical coherence tomography provides full thickness imaging of the chorioretinal complex

Egidijus Auksorius, Dawid Borycki, Piotr Wegrzyn, Bartosz L. Sikorski, Kamil Lizewski, Ieva Zickiene, Mounika Rapolu, Karolis Adomavicius, Slawomir Tomczewski, and Maciej Wojtkowski

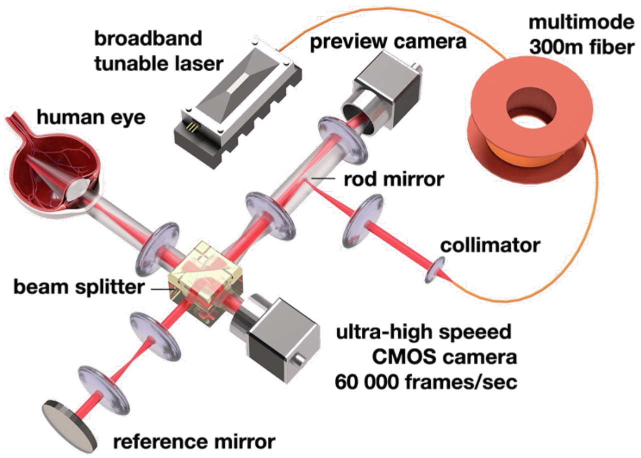

Figure S1. Optical scheme of STOC-T system. STOC-T is based on a simple interferometer design that incorporates an ultrahigh-speed camera, a tunable laser source for recording retinal volumes (in the form of multispectral interferograms) and a 300 meter-long multimode fiber with a 50 µm core that produces ~250 well-defined spatial modes (TEMs), which illuminate a sample one-by-one within a couple of nanoseconds. The differences between the illumination and backscatter in the pupil is also exploited to implement a preview camera that provides video-rate axial images of the retina for focus finding. It detects the backscattered signal from the retina that is lost in a conventional interferometer design. Related to **STAR Methods – STOC-T setup**.

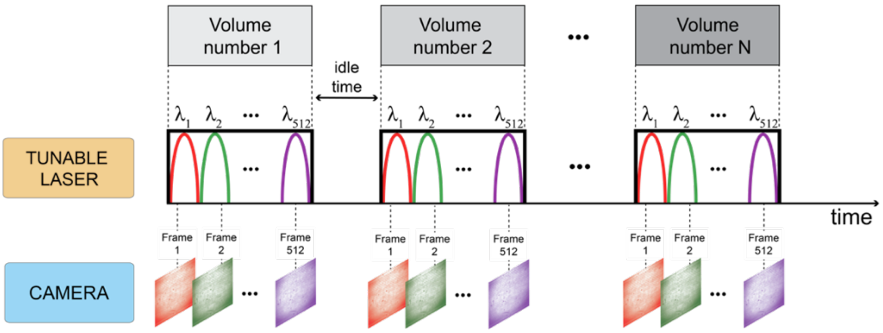

Figure S1. STOC-T data acquisition scheme. The wavelength of the tunable laser is changed from λ_1 to λ_{512}. At each instantaneous wavelength, we capture a corresponding two-dimensional interferogram using the camera. We repeat this process N times to obtain the dataset that is processed digitally. Related to **STAR Methods – Data analysis.**

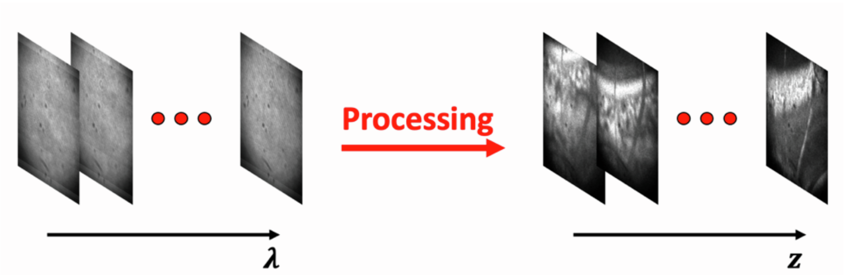

Figure S2. A representative illustration of the raw (left) and processed data (right). Related to **STAR Methods – Data analysis**.

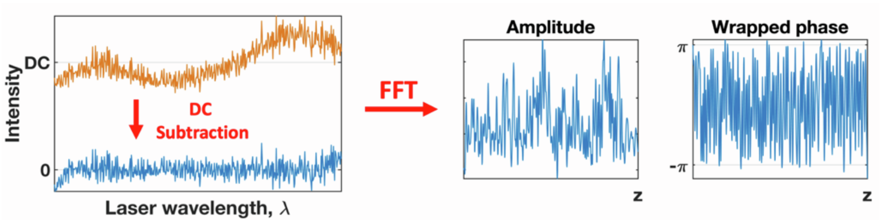

Figure S3. Initial step in signal processing. The DC-subtracted signal of length N (2048 after zero-padding) is transformed with Fast Fourier Transformation to achieve amplitude and phase that encode depth-information about the sample. Related to **STAR Methods – Data analysis**.

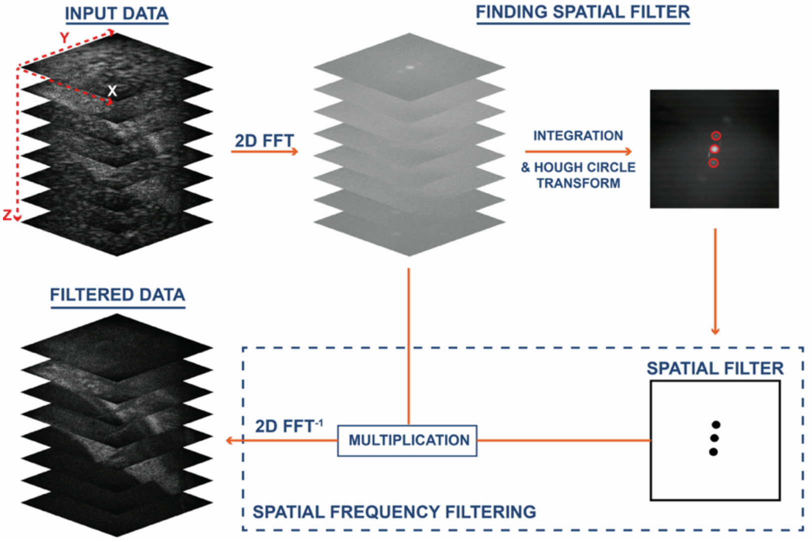

Figure S4. Spatial frequency filtering suppresses low-frequency speckles from the multi-mode fiber. Each en face plane of the input data is 2D Fourier transformed to achieve a 3D stack of the spatial spectra. This stack is then integrated along Z to obtain the 2D image, which is subsequently processed with Hough Circle Transform to find circles. The resulting rings are employed to construct the mask multiplied by each XY plane in the spatial spectra stack. The resulting data is inverse Fourier-transformed to obtain filtered volume. Related to **STAR Methods – Data analysis**.

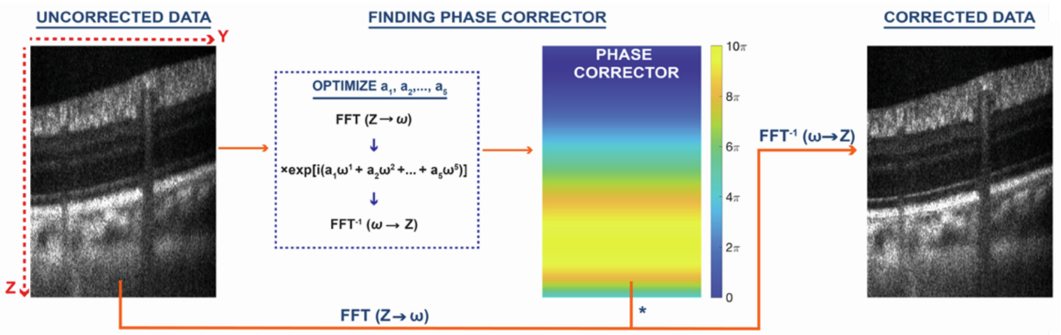

Figure S5. Correction of axial motion and dispersion. Axial motion and dispersion corrections improve axial resolution to resolve retinal layers better. The uncorrected B-scans (YZ) planes are iteratively corrected in the reciprocal space by changing the adjustable parameters $a_1, a_2, \ldots, a_5$ until the image sharpness metric is optimized. The resulting phase corrector is then applied to all B-scans. Related to **STAR Methods – Data analysis.**

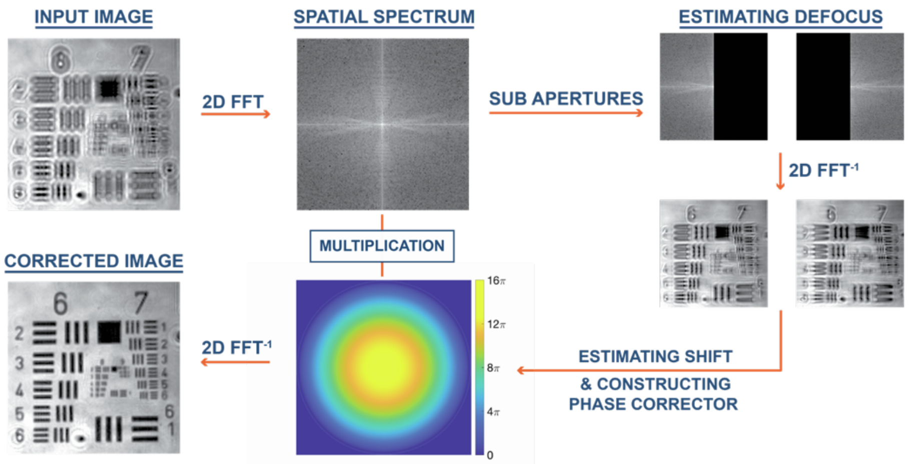

Figure S6. Estimation of defocus via split aperture method. To correct for defocus, we used the split aperture approach. For selected depth or Z range, we estimate, depth-resolved defocus parameter, $d(Z)$. To do so, we start with an *en face* image, which is then converted to the spatial frequency domain using 2D FFT (Fig. S7). Afterward, we split the Fourier spectra into two equally sized sub-apertures, and calculate their inverse 2D FFT. We then estimate the shift between resulting images, which is directly proportional to the defocus parameter. We perform this process for other depths to obtain $d(Z)$. As for some depths, the estimated values might deviate from the linear trend, we reject outliers. Then we fit $d(Z)$ to a line, $D(Z) = aZ + b$. Given the parameters $a, b,$ we then constructed the phase corrector, $P(z) = \exp[iD(z)Z_2^0]$, where Z_2^0 denotes the defocus Zernike polynomial. We repeat this process for all other volumes in the dataset. Then, we average parameters a and b to obtain the best possible estimate for the phase corrector. In the last step, the phase corrector is applied to all volumes in the dataset. Related to **STAR Methods – Data analysis**.

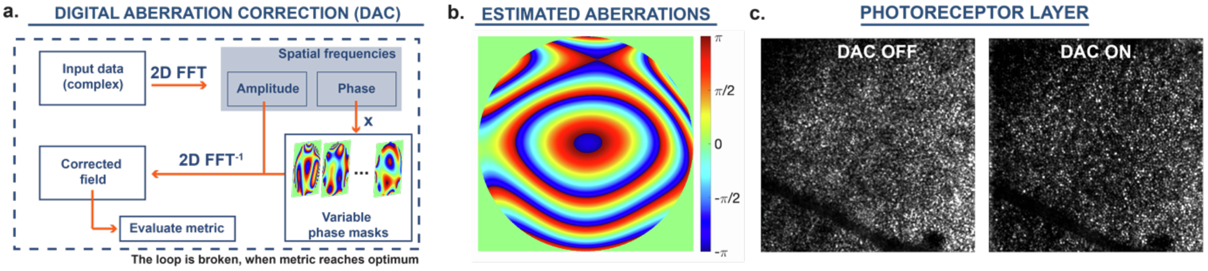

Figure S7. Digital aberration correction. (a) The sketch of the numerical algorithm*:* the input en face complex image, $U(X,Y,Z)$ is 2D Fourier-transformed to yield $\widetilde{U}(k_x,k_y,Z)$. Then $\widetilde{U}(k_x,k_y,Z)$ is multiplied by the variable phase mask, $P_a(k_x,k_y) = \exp\left[i\sum_n\sum_m \alpha_{nm}Z_n^m(k_x,k_y)\right]$, where α_{nm} are adjustable parameters (or Zernike weights) and $Z_n^m(k_x,k_y)$ denote the Zernike polynomials. The resulting product $\widetilde{U}(k_x,k_y,Z)P_a(k_x,k_y)$ is 2D-inverse-Fourier transformed to obtain phase-corrected data, $U_{corr}(X,Y,Z)$. We then calculate the image sharpness metric (entropy) using the intensity of the corrected image, $|U_{corr}(X,Y,Z)|^2$. This process is continued until we optimize the metric. (b) Representative phase mask of the first 21 Zernike polynomials (excluding piston, tilt, and tip). (c) Reconstruction of photoreceptor cone mosaic. Related to **STAR Methods – Data analysis.**

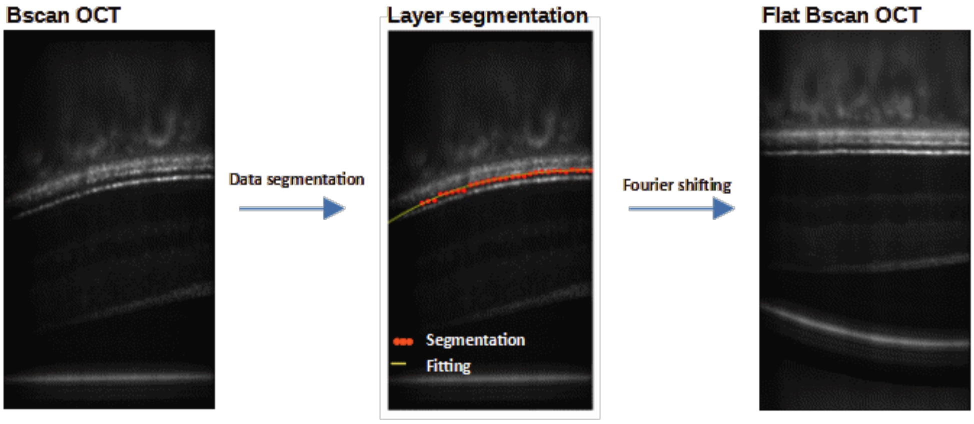

Figure S9. The scheme of the volume flattening method*.* A polynomial surface was fitted to the manually marked RPE layer on a selected number of b-scans (10 XZ and 10 YZ) using the least squares method. This surface was then used to shift all the A-scans in the volume axially, using the Fourier shift theorem, so that the RPE layer is at one depth. Related to **STAR Methods – Data analysis**.

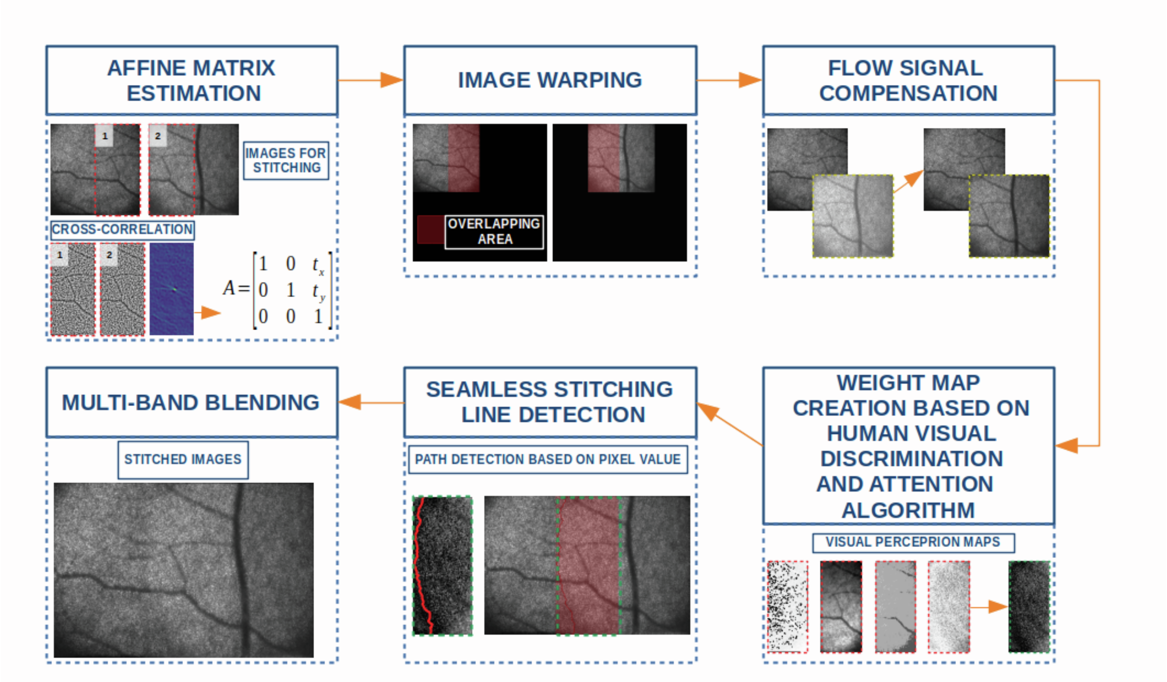

Figure S8. The scheme of the image stitching algorithm. Related to **STAR Methods – Data analysis**.

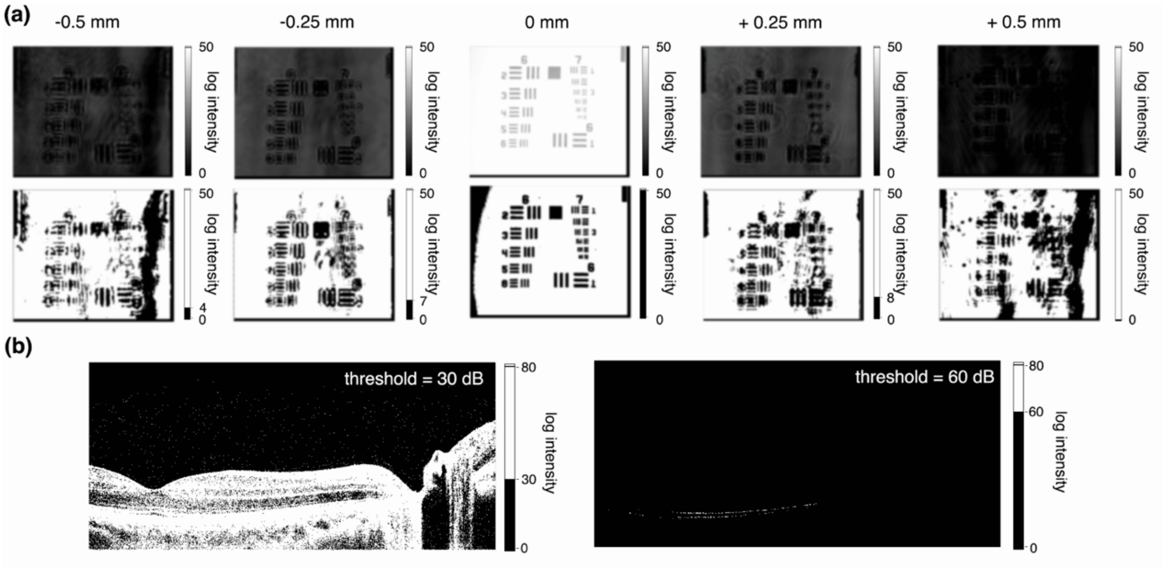

Figure S11. Analysis of the dynamic range of scanning OCT. (a) Observation of surface brightness changes of a USAF test target reconstructed by scanning OCT with defocus; data presented on a logarithmic scale. The bottom row shows binary images with thresholded values quantitatively showing the brightness level of the reconstruction. The obtained dynamic range is about 60dB with intensity drops for $\pm$ 0.25 mm of about 43dB and for $\pm$ 0.5 mm of over 45 dB. The laboratory OCT scanning system was an SdOCT instrument with a commercial spectrometer from Wasatch Photonics. The SdOCT system was set to the same level of lateral resolution and used the same 10x objective (NA=0.25) as the STOCT system. Sensitivity roll-off was accommodated in this experiment by always making measurements for the same optical path difference. (b) binarization of retinal OCT data showing typical dynamic range of about 25-30dB in Scanning OCT reconstructions. Related to **Figure 2.**

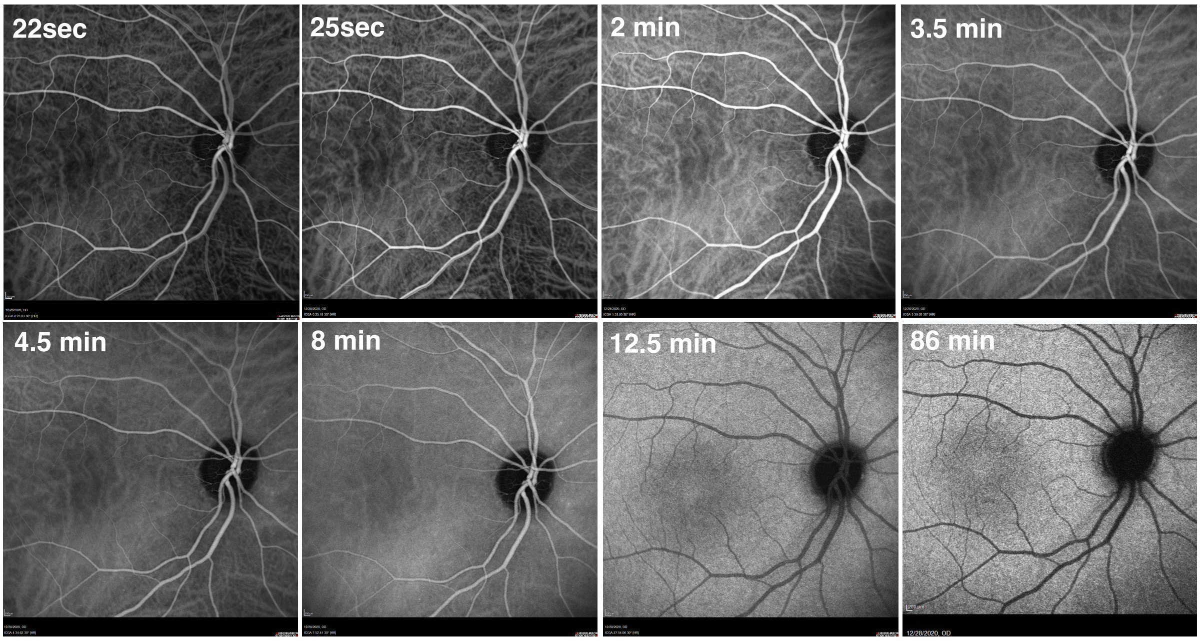

Figure S12. ICG angiography. ICG images acquired at different points in time does not reveal choroidal microsctructure. Related to **Figure 6.**

Calculation of maximum permissible radiant exposures MPH_c and maximum permissible radiant powers $MP\Phi$:	
<u>Cornea:</u>	***Case 1,* t >10 s**<br>**MPH_{Co} = 4 W/cm²**<br>Power density of light beam illuminating the cornea during the STOC-T measurements:<br>PD_{cornea} = 5mW / 0.02 cm²= **250 mW/cm²**
	***Case 2,* Worst case scenario (alignment of volunteer's head) with 0.1mm spot on the cornea for t >10 sec:**<br>$MP\Phi_{cornea}= MPH_{Co} \times S_{cornea}$ => 4 W / cm² x 7.85 x 10^{-3} cm² ~ 32 x 10^{-3} W = <u>32 mW</u><br>Assuming linear scaling of MPE with the spot diameter:<br>**$MP\Phi_{cornea, 0.1mm}$~ 3.2mW**
	***Case 3,* Worst case scenario (alignment of volunteer's head) with 0.1mm spot on the cornea for t =1 sec:**<br>MPH_{Co}= 25 x $t^{-0.75}$ W cm^{-2}=25 W cm^{-2}<br>$MP\Phi_{cornea}= MPH_{Co} \times S_{cornea}$ => 25 W / cm² x 7.85 x 10^{-3} cm² ~ 196 x 10^{-3} W = <u>196 mW</u><br>Assuming linear scaling of MPE with the spot diameter:<br>**$MP\Phi_{cornea, 0.1mm}$~ 20mW**
<u>IRIS:</u>	***Case 1,* t >10 s**<br>**MPH_I =** 5x 0.2 *C_A = 1.58 x 0.2 [W/cm²] ~ **1.6 [W/cm²]**<br>Experiment: PD_{iris} = 5mW / 0.02 cm²= **250 mW/cm²**
	***Case 2,* Worst case scenario (alignment of volunteer's head) with 0.1mm spot on the iris for t >10 sec:**<br>$MP\Phi_{IRIS} = MPH_I \times S_{IRIS}$ = 1.6 W /cm² x 9.62x10^{-2} cm² ~ <u>155 mW</u><br>Assuming linear scaling of MPE with the spot diameter:<br>**$MP\Phi_{iris, 0.1mm}$~ 4.4mW**
	***Case 3,* Worst case scenario (alignment of volunteer's head) with 0.1mm spot on the cornea for t =1 sec:**<br>**MPH_I =** 5x 1.1 *C_A = 1.58 x 1.1 [W/cm²] ~ **9 [W/cm²]**<br>$MP\Phi_{IRIS} = MPH_I \times S_{IRIS}$ = 9 W /cm² x 9.62x10^{-2} cm² ~ <u>83 mW</u><br>Assuming linear scaling of MPE with the spot diameter:<br>**$MP\Phi_{iris, 0.1mm}$~ 8mW**
<u>RETINA</u>	t >10 s -> $MPH_C = 1.8 \times C_A \times C_E\ T_2^{-0.25} \times 10^{-3}$[W/cm²]<br><br>$C_{A-800nm}=10^{0.002(800-700)}$=1.58<br>$C_E= \alpha / \alpha_{min}$ =100mrad/1.5mrad =66<br>Size of extended retinal illumination:1.7 mm of diameter (0.85 mm radius)<br>α=2xarc tan(0.85/17) =2x0.05 rad = 0.1rad =100 mrad<br>α_{min} = 1.5 mrad, α_{max} = 100 mrad => $\alpha=\alpha_{max}$<br>T2 =10x10 $^{(\alpha-1.5)/98.5}$=10x10¹=100sec = >$T_2^{-0.25}$= 0.32<br>P (pupil factor) =1<br><br>**MPH_c=1.8x1.58x66x0.32 x10^{-3}[W/cm²] = 60 [mW/cm²]**<br>$MP\Phi$=60[mW/cm²]* 0.385cm²= <u>22mW</u>

Table S1. Detailed safety analysis for STOC-T. Related to **STAR Methods – Eye Safety**.
